# Photo‐Induced Cracking in a Bi‐Component Molecular Solid: Capturing Structural Intermediates

**DOI:** 10.1002/asia.202401518

**Published:** 2025-02-26

**Authors:** Mollah Rohan Ahsan, Manish Kumar Mishra, Arijit Mukherjee

**Affiliations:** ^1^ Department of Chemistry BITS Pilani Hyderabad Campus Shamirpet Jawhar Nagar, PIN 500078; ^2^ Physical and Materials Chemistry Division CSIR-National Chemical Laboratory Homi Bhabha Road, Pashan Pune India; ^3^ Academy of Scientific and Innovative Research (AcSIR) Ghaziabad 201002 India

**Keywords:** crystal engineering, bi-component solid, [2+2] photodimerization, photoinduced cracking, single-crystal-to-single crystal transformation

## Abstract

A photo‐responsive bi‐component solid is designed based on robust large synthons. The study not only provides a template‐based approach for a system that was difficult to photo‐dimerize, but the designed solid also leads to photo‐induced cracking upon photodimerization. The delicate structural design helps in capturing the structural intermediates, which reveals a possible reorientation of the photodimer within the crystal to maintain structural integrity.

## Graphical abstract

The concept of Long‐range Synthon Aufbau Modules (LSAM) is used to design photo‐responsive bi‐component solids. The design strategy is primarily based on tuning the structural parameters of a robust large synthon that leads to [2+2] photodimerization along with photo‐induced cracking. Captured structural intermediates reveal a possible reorientation of the [2+2] photodimer within the crystal.

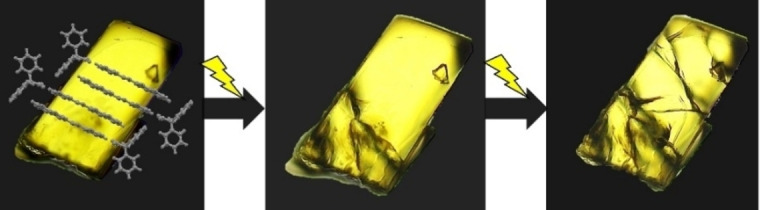

## Introduction

Design and synthesis of stimuli‐responsive materials have become a frontier research area in chemistry and material science.[^1^, ^2^] Since crystal engineering has emerged as a discipline that provides reliable strategies for designing organic solids, it intends to play a significant role to the above objective.[^3^, ^4^] While the design of these materials can adopt variegated approaches,[^5^, ^6^, ^7^] photo salient materials have garnered more attention mainly due to their non‐contact and non‐invasive nature.^[8]^ Recent reports have shown various instances of photo‐induced mechanical effects such as cracking,^[9]^ bending^[10]^ or jumping.[^11^, ^12^] Many of these effects result from photo‐induced reactions,^[9]^ indicating a possible correlation between structural changes at the molecular level and macroscopic response in crystals. This highlights the exceptional potential crystal engineering may offer in designing these solids.

[2+2] Photodimerization of molecular solids is generally explained by the topochemical principle where the olefin groups within ~4.1 Å undergo photoinduced structural transformation to cyclobutane derivatives.[^13^, ^14^, ^15^] Among several photomechanical effects associated with photodimerization, the cracking of single crystals, sometimes followed by jumping, has remained a general observation, with examples primarily based on coordination polymers[^16^, ^17^] and a few cases in organic systems.[^12^, ^18^] Systematic exploration of such effects in molecular crystals has remained difficult due to the structural disintegration that generally follows in such cases. Since the changes in such transformations are often analyzed by isolating a recrystallized product and subsequent crystal structure analysis, the role of structural intermediates in such cases has remained unclear, charting a research gap between photo‐induced effects and single‐crystal‐to‐single‐crystal (SC‐SC) transformation, especially for molecular solids.^[9]^

Here, we report a bicomponent molecular solid that exhibits photo‐induced cracking while undergoing [2+2] cycloaddition reaction under visible and UV light. The system was carefully designed using crystal engineering principles starting from structures previously reported by our group.[^19^, ^20^] The photo‐induced crack generations were slow enough to capture the structural intermediates through SC‐SC transformations.

**Scheme 1:**
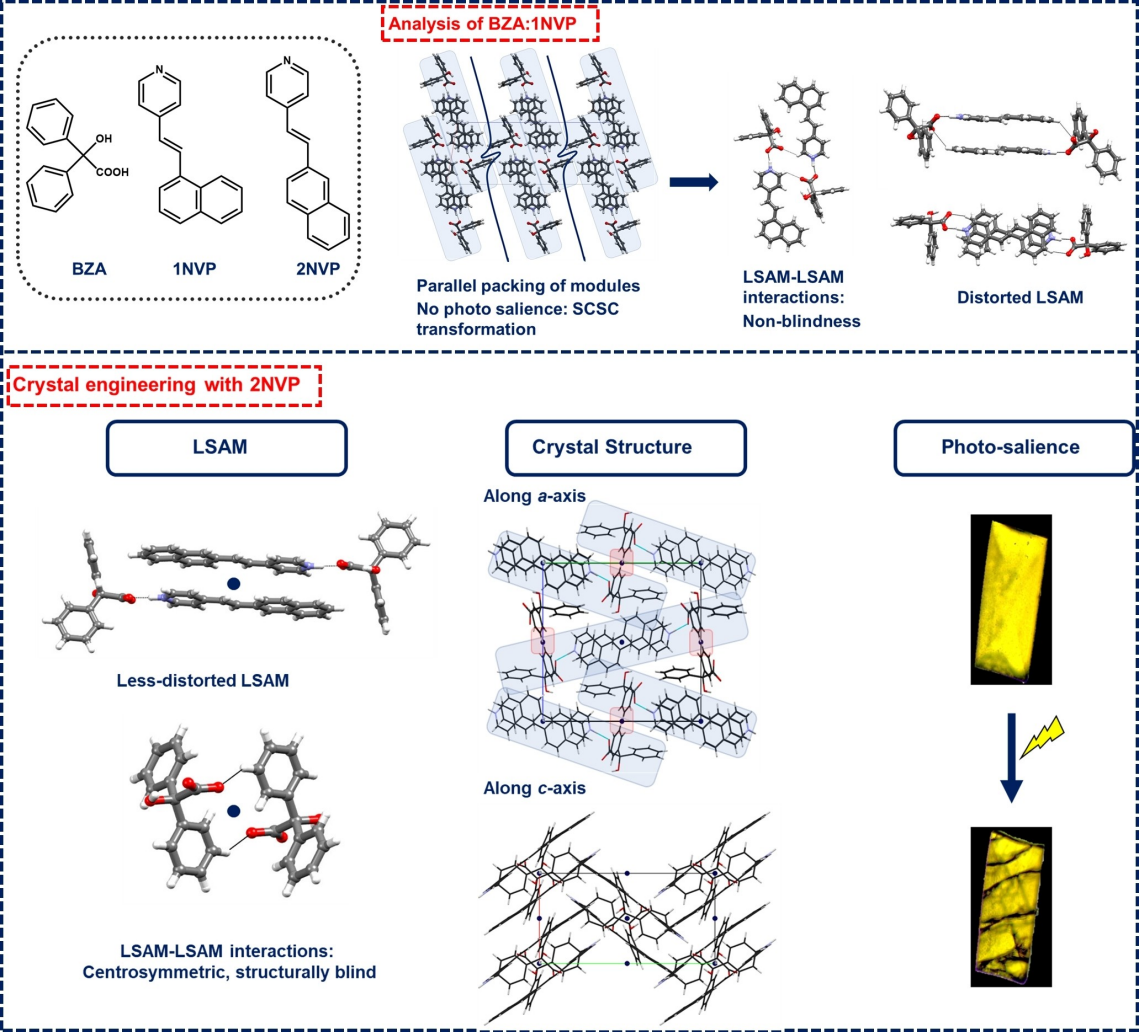
Crystal engineering strategy toward photo‐induced cracking. LSAMs are shown in blue patches. The red patches highlight the structural blindness among LSAMs in BZA‐2NVP.

In a recent study, we reported the occurrence of SC‐SC transformation in Benzilic acid (BZA) and (*E*)‐4‐(2‐(naphthalene‐1‐yl) vinyl) pyridine (1NVP) salt without any visible photomechanical effect.^[19]^ These observations were contrary to what was observed in BZA: ((*E*)‐4‐(3,5‐dichloro styryl)pyridine) (DCP) bi‐component solids, which underwent photoinduced solid to‐liquid transformation.^[20]^ Since both these solids are sustained through a similar large synthon, the packing differences in these solids arise due to a slight distortion of the large synthon in BZA‐1NVP, causing differences in Long‐range Synthon Aufbau Modules (LSAM).[^21^, ^22^] The distortion of large synthon in BZA‐1NVP was mainly due to C−H⋅⋅⋅O interactions between BZA molecules and strong π⋅⋅⋅π interactions among the 1NVP molecules that led to a parallel LSAM in BZA‐1NVP. In this context, we envisaged that a more linear naphthyl derivative would reduce large synthon distortion and eventually form an LSAM incorporating more structural insulation among the components (Scheme 1). Hence, (*E*)‐4‐(2‐(naphthalene‐2‐yl)vinyl)pyridine (2NVP) was chosen as a coformer with BZA for this study. 2NVP does not undergo photodimerization in its native state.^[23]^ A template‐based approach was adopted before using thiourea as a template to make it photoreactive. Although 2NVP formed a cocrystal with thiourea, the resulting solid was not photoreactive due to not meeting the topochemical criteria.^[24]^ An inorganic salt‐based approach was coined later, which showed that 2NVP can become photoreactive only in a hydrated salt, while the native salt remained unreactive.^[23]^ Since incorporating solvents in any crystal structure is unpredictable,^[25]^ this solid possibly lacked appropriate design elements. Therefore, the first question we sought in this study was *whether BZA can act as a suitable template for the photodimerization of 2NVP*. Since the resulting bi‐component solid also showed photomechanical responses in an SC‐SC fashion, we tried to obtain single‐crystal structures of intermediate stages that shed light on the mechanism and showed the gradual transformation of olefins to respective photo products.

**Figure 1:**
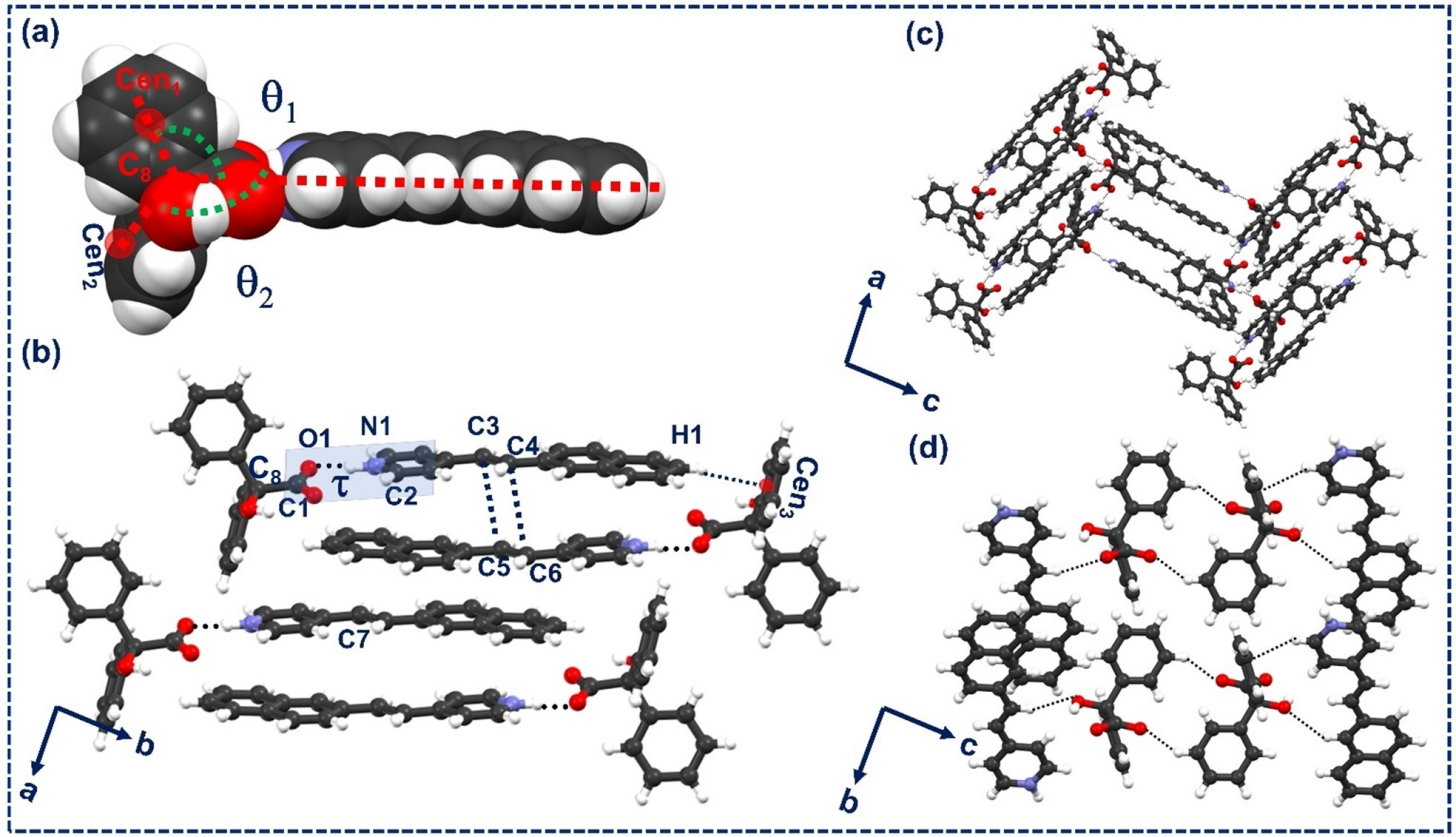
(a) Primary synthon formed between BZA and 2NVP. θ_1_ and θ_2_ depict the angles between phenyl rings in BZA and 2NVP. (b), (c), (d) packing diagrams of BZA‐2NVP, viewed along *c*‐, *b‐*, and, *a*‐ axes, respectively.

**(a) Structural description of BZA‐2NVP**: The binary salt of BZA and 2NVP was obtained as yellow‐colored crystals through liquid‐assisted grinding using acetonitrile followed by solvent evaporation from the same solvent. It was also obtained by cooling crystallization from selective ionic liquids, such as 1‐Ethyl‐3‐methylimidazolium bis(trifluoromethyl sulfonyl)imide ([Emim]^+^[NTF_2_]^−^) and 1‐(2′‐hydroxyethyl)‐3‐methylimidazolium bis(trifluoromethyl sulfonyl)imide ([OH‐Emim]^+^[NTF_2_]^−^).^[26]^ Single‐crystal X‐ray analysis showed that the desired binary solid was packed in the monoclinic space group, *P*2_1_/c, with Z′=1. In the crystal structure, 2NVP interacts with BZA through charge‐assisted O^−^⋅⋅⋅H- N^+^ (2.547(2) Å) hydrogen bonds (Figure 1b). The angle between 2NVP and the phenyl rings of BZA (θ_1_=118.70°, θ_2_=102.93°), and the torsional angle around the respective carboxylate‐pyridinium synthon (τ=19.1(2)°) seem sufficient to form a modular geometry (Table 1). These primary synthons are related by an inversion center and lead to the formation of a large modular synthon that packs the 2NVP molecules in a *head‐to‐tail (ht)* fashion within 3.631(3) Å (Figure 1b). Since it satisfies Schmidt's topochemical criteria (<4.1 Å),^[13]^ we envisaged a successful photoreaction between 2NVP molecules leading to a *ht‐*photoproduct. The parallel alignment (Γ=0) of olefinic double bonds of 2NVP molecules further supports the above hypothesis.^[14]^ Since inter‐module separation lies ∼7.078(4) Å, the desired photoreaction may occur only within the module (Table 1).

**Table 1:** Geometrical parameters associated with structures I, II, III, and IV.^[27]^

Structure	d_1_(Å)	θ_1_ (°)	θ_2_ (°)	τ (°)	d_2_(Å)	d_3_(Å)	d_4_(Å)	α (°)	Γ
Parameter	**O_1_**−**N_1_**	**Cen_1_**−**C_8_**−**H_1_**	**Cen_2_**−**C_8_**−**H_1_**	**C_1_**−**O_1_**−**N_1_**−**C_2_**	**C_3_**−**C_5_**	**C_5_**−**C_7_**	**H_1_**−**Cen_3_**	**C_3_**−**C_5_**−**C_6_**	**C_3_**−**C_4_**−**C_5_**−**C_6_**
**I**	2.547 (2)	118.70	102.93	19.1(2)	3.631(3)	7.078(4)	2.775	106.4(2)	0.00 (8)
**II**	2.548 (4)	118.16	102.90	17.8(5)	3.626(7)	7.069(8)	2.731	105.9(3)	0.00 (2)
**III**	2.533 (6)	116.10	106.24	11.4(6)	1.57(1)	7.74(1)	2.813	89.8(7)	0.00 (7)
**IV**	2.528 (6)	116.03	107.34	14.1(6)	1.568(9)	7.58(1)	2.658	90.3(5)	0.00 (5)

**Figure 2:**
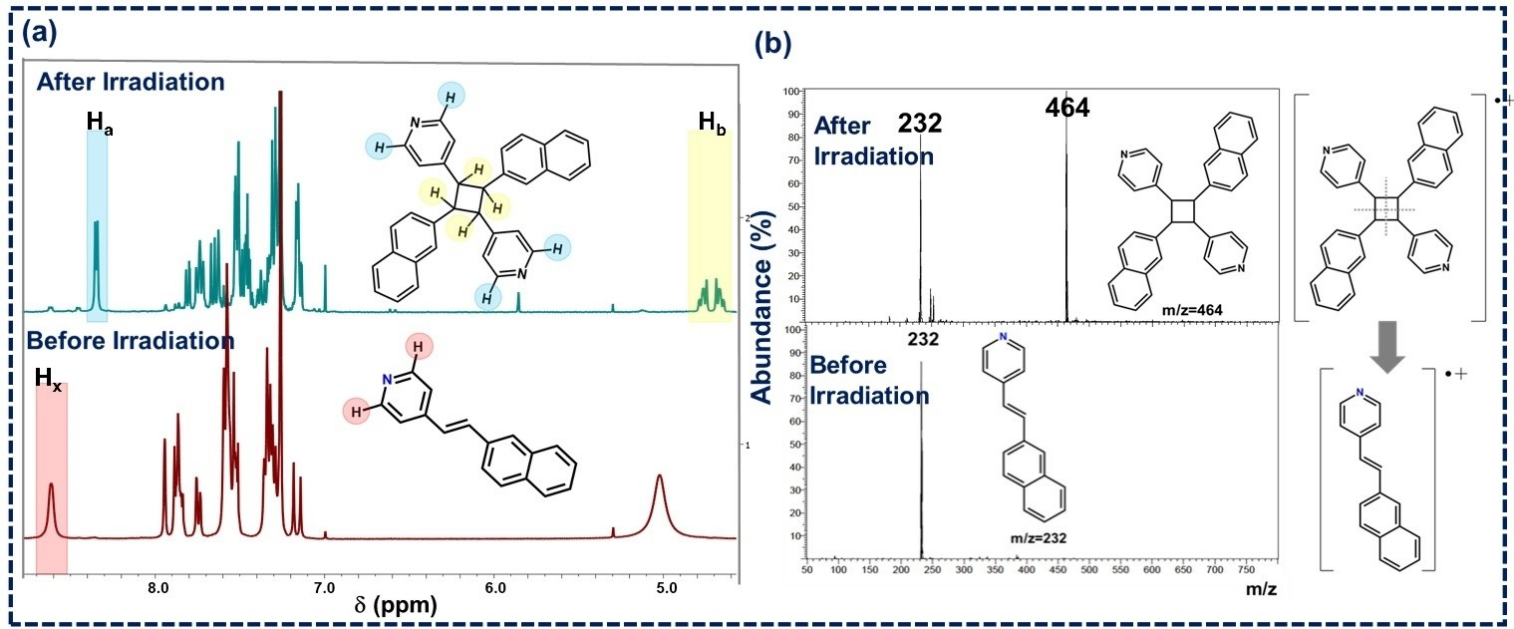
(a) ^1^H NMR of native and irradiated sample under broadband UV light. Note the appearance of a new peak (Ha) in ^1^H NMR depicting the formation of the photodimer. (b) LC–MS spectra of before and after irradiated sample under broadband UV light.

**(c) Photo chemical behavior and structural analysis of the dimerized sample**: Crystalline powder of BZA‐2NVP was irradiated under broadband UV radiation for 10 hours. The irradiated powder sample was subsequently dissolved in CDCl_3_, and the % conversion to photoproduct was analyzed by ^1^H NMR spectroscopy. The disappearance of the pyridyl proton peaks at δ=8.66 ppm (H_x_) and new proton peaks at δ=8.34 ppm (H_a_) indicates a photo dimer formation (Figure 2a). In addition, the integration of the protons attached to the formed cyclobutane ring (H_b_, δ=4.64–4.82 ppm) also matched with the pyridyl proton (H_a_). In addition, the integration of the protons attached to the formed cyclobutane (H_b_) ring also matched with the pyridyl proton (H_a_) (at δ=8.34 ppm). The compound was also dimerized under visible light, as confirmed through the peak at m/z= 464 in the respective LC–MS spectrum (ESI‐S10).

**Figure 3:**
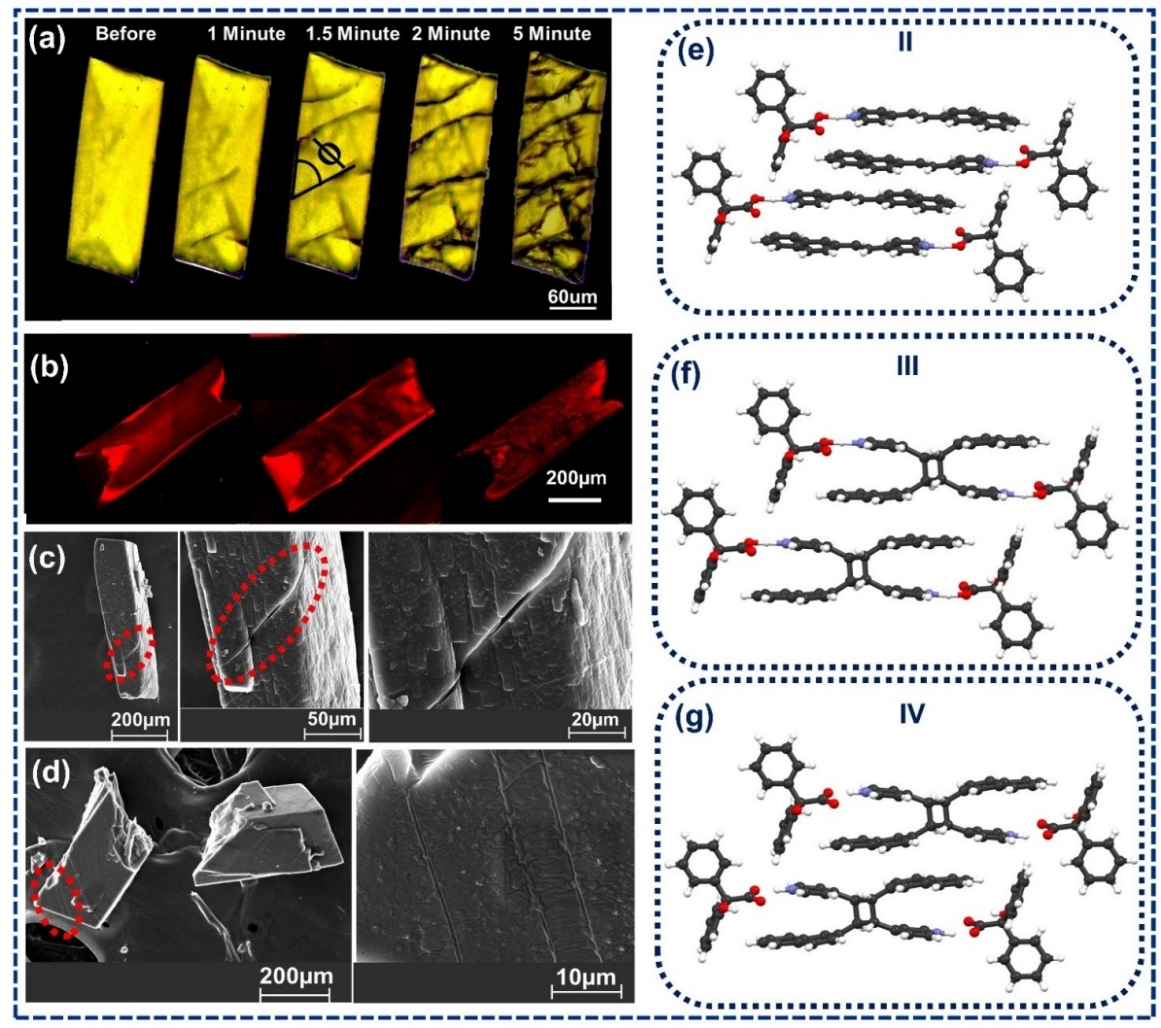
(a) Microscopic, (b) Confocal images of crystal cracking after irradiation under visible light. (c) SEM images of the cracks being generated on the crystal surface and (d) SEM images of disintegrated crystals. Packing diagrams of (e) **II**, (f) **III**, and (g). **IV**.

While the single crystals of **I**, as obtained from the solution crystallization of powdered samples of the native compound, were studied under an optical microscope, some cracks were observed (Figure 3a). Cracks were also observed when the crystals were placed under a confocal microscope (λ_ex_=420 nm) (Figure 3b). Since the crack formation under an optical microscope indicates the feasibility of photoreaction under visible light, obtained single crystals were left in sunlight, and the data was collected for three intermediate stages (**II**, **III**, and **IV**).

In **II**, structural refinement suggests ∼20 % cyclobutane formation. Positive expansion along the *a* and *c*‐axes (0.094 % and 0.67 %) and a negative expansion along the *b‐*axis (−0.36 %) were observed in **II**. The relative volume of **II** changed only slightly (∼0.5 %) compared to **I** (Table 2). A slight change in torsional angle for carboxylate–pyridinium synthon, however, suggested partial dimerization as the respective torsional angle in **II** was decreased to τ=17.8(5)° from τ=19.1(2)° in **I** (Figure 3e).

**Table 2:** Change in unit cell parameters during photodimerization.

Parameter	**I**	**II**	**III**	**IV**
**a/Å**	8.6372(8)	8.6453(16)	8.7894(18)	8.642(7)
**b/Å**	16.4566(12)	16.398(3)	16.182(4)	15.920(13)
**c/Å**	16.8871(13)	17.001(3)	17.390(4)	17.265(12)
**α/°**	90	90	90	90
**β/°**	103.164(3)	102.967(6)	101.561(5)	101.907(18)
**γ/°**	90	90	90	90
**V/Å^3^**	2337.24	2348.7	2423.2	2324.22
**Relative change** **(ΔV%)** **compared to I**	–	0.49	3.68	−0.56

In **III**, the structural refinement shows ∼55 % cyclobutane formation. A positive expansion along the *a* and *c*‐axes (1.76 % and 2.98 %) and a negative expansion along the *b*‐axis (−1.67 %) were observed, and the overall cell volume was increased by 95.96 Å^3^ (3.68 %) (Table 2). The torsional angle between the carboxylate and pyridinium (τ) further decreased to 11.4 (6)° (Figure 3f).

In **IV**, the structural refinement indicates ∼80 % cyclobutane formation. From the analysis of cell parameters, a positive expansion along the *a* and *c*‐axes (0.056 % and 2.24 %), and a negative expansion along the *b*‐axis (3.26 %) were delineated while the cell volume was decreased by 0.56% compared to **I** (Table 2). The torsional angle between carboxylate–pyridinium synthon was ∼14.1(6)°. This value lies between the values observed for **I** and **III** (Figure 3g).

In most of the reported cases of photosalient crystals, the volume change is the main reason behind the macroscopic effect, where the volume change generally ranges between 7–20 % in most of the systems,[^16^, ^17^, ^28^, ^29^, ^30^] with only a few cases where volume change upon dimerization was found to <7%.[^9^, ^11^] In this case, the minimal volume change upon dimerization, as observed in **I**–**IV,** indicates small changes in the reactive geometry (ESI‐S6).

**(c) Photomechanical behavior of the structure**: The photoirradiation of single crystals of BZA‐2NVP led to diagonal cracks on the prominent crystal face. The distance between the cracks varied between 47–75 μm with an average separation of 62 μm (ESI‐S5d), and the average crack angle observed was φ=60° (ESI‐S5d). More importantly, the crystals didn't lose their crystallinity while the cracks were generated. A face indexing of **I** showed the observed major face was (011) (ESI‐S5a), which correlated well with the BFDH calculation (ESI‐S5b).^[31]^ The indexing of PXRD data indicates (011) is also the major face in the bulk sample, indicating that photodimerization may happen through a similar mechanism in single crystals and the bulk (ESI‐S6). Initially developed cracks are generally parallel to (100) planes, as shown in (ESI‐S5b). When the average cracking distance was correlated with crystal structure, it suggested that around 17*10^4^ molecules were dimerized to generate the strain that caused the cracking (ESI‐S5e). The intensity mapping of confocal microscopic images collected for single crystals of **I** and after exposure under a 420 nm laser (Figure 3b) showed decreased intensity around the cracks. This indicates a heterogeneous propagation of the reaction that possibly generates cracks in the crystals. The cracks were also analyzed through SEM (Figures 3c and 3d).

**Figure 4:**
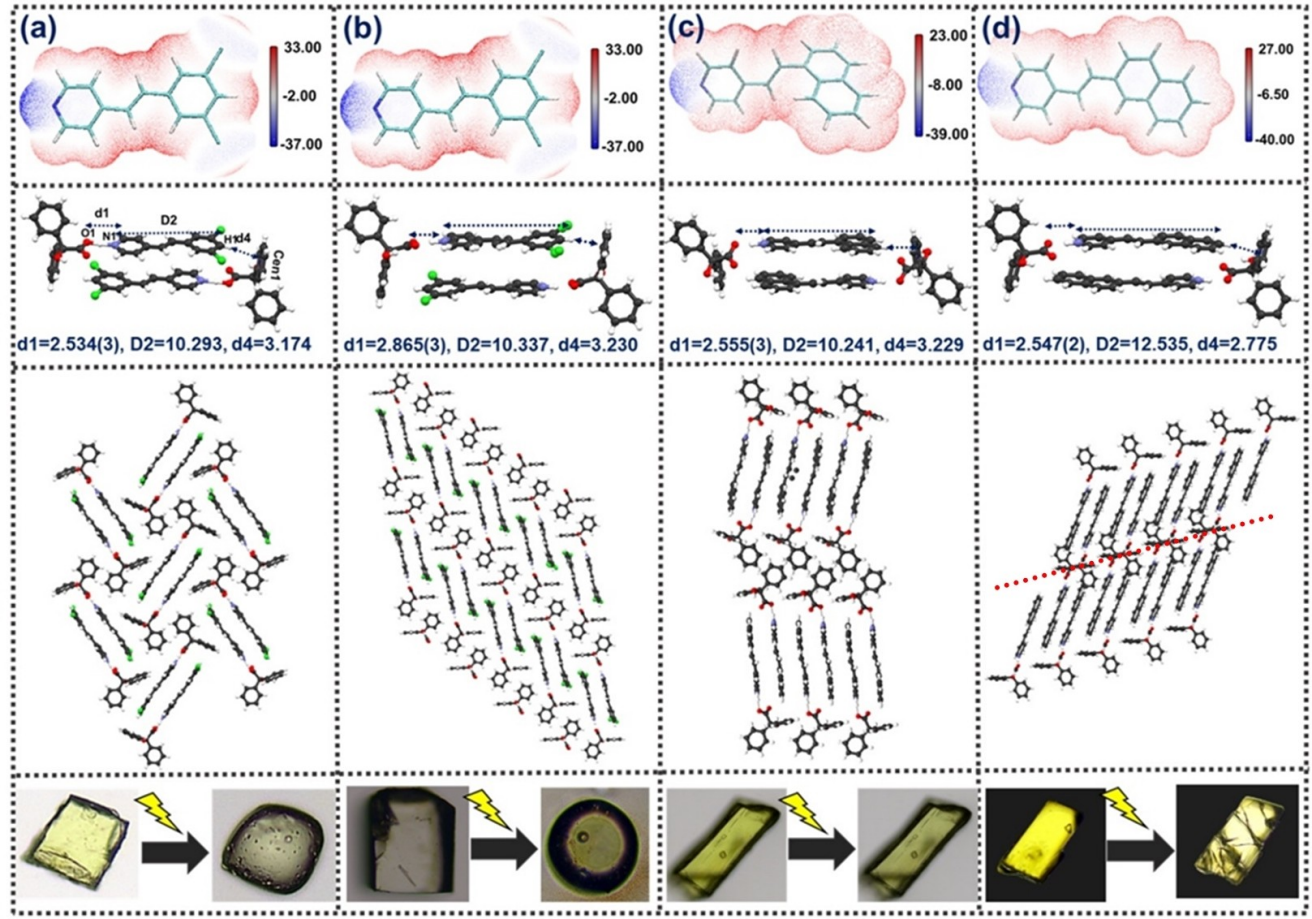
ESP, packing, and photomechanical behavior comparison of (a) BZA‐DCP Form I, (b) BZA‐DCP Form II, (c) BZA‐1NVP, and (d) BZA‐2NVP (The dotted‐red represents slip‐planes in the structure).

**(d) Structural comparison of photoreactive multicomponent solids using BZA template**: BZA has proven to be a robust template for controlling photodimerization in a modular fashion in 4‐styrylpyridine derivatives. The photomechanical responses in these systems, however, have differed drastically. During the photoreactions, the BZA‐DCP system was sustained through a solid‐to‐liquid transition, and BZA‐1NVP underwent an SC‐SC transformation without any visible changes (Figure 4a, 4c).[^19^, ^20^] Since the current system of BZA‐2NVP seems to lie between these two structural extremes, leading to cracks (Figure 4d), we sought a structural investigation between BZA‐2NVP with BZA‐DCP and BZA‐1NVP. The differences in photomechanical responses between BZA‐1NVP and BZA‐DCP can be explained by the difference in torsional angles pertaining to carboxylate‐pyridinium synthon (τ=41.7(2)° in BZA‐1NVP as compared to (τ=3.7(3)° in BZA‐DCP‐Form I). The difference in primary synthon controls the organization of LSAM, eventually leading to different crystal packing in these two solids.

Compared to the above, the differences become subtle between BZA‐DCP and BZA‐2NVP due to the similarity in respective primary synthons. The primary modules in both cases remain similar as well. In the BZA‐DCP (Form I) structure, the modules are packed in a herringbone fashion, leading to a solid‐to‐melt transition. Recently, a second form of BZA‐DCP (BZA‐DCP‐Form II) was obtained in our lab through solution evaporation from ethanol (Figure 4b). This robust modular synthon, as observed in BZA‐DCP Form I, was also observed between BZA‐DCP Form II. In addition to the presence of disordered DCP molecules in the crystal structure, it differs from Form I in the LSAM formation. This form also undergoes a solid‐to‐liquid transformation upon photoirradiation, as observed in Form I. While the solid‐to‐liquid melting behavior in BZA‐DCP Form I can be explained through the herringbone packing, the melting upon irradiation in the case of BZA‐DCP form II highlights the role packing between two BZA molecules through C⋅H⋅⋅⋅π interaction, leading to a lack of slip planes. The question, therefore, remains regarding structural parameters that contribute to the differences in photomechanical behavior in BZA‐DCP (Form II) and BZA‐2NVP.

To investigate this, an electrostatic potential (ESP) calculation was carried out on these coformers that suggests the pyridine nitrogen has less negative potential (−37 kcal/mol) compared to 2NVP (−40.05 kcal/mol), indicating the stronger hydrogen bonding between BZA and 2NVP compared to that in BZA‐DCP. These two molecules also differ in the molecular length; While the molecular length (D2) of DCP is ∼10.293 Å, the D2 for 2NVP is ∼12.535 Å, helping the latter to form a stronger C−H–π contact (δ4=3.697 Å) between BZA and 2NVP.

If BZA‐NVP is closely compared with BZA‐DCP (Form II) in terms of LSAMs, it can be observed that the modular synthons as formed in BZA‐2NVP are more robust due to stronger carboxylate pyridinium synthon, π–π stacking, and C−H⋅⋅⋅O interactions. This leads to more insulated packing of large synthons in BZA‐2NVP as compared to BZA‐DCP or BZA‐1NVP, eventually leading to directional slip planes in the BZA‐2NVP system.

## Conclusions

This study is focused on photodimerization and associated photomechanical effects of a binary solid of Benzilic acid (BZA) and ((E)‐4‐(2‐(naphthalene‐2‐yl)vinyl)pyridine) (2NVP). 2NVP is photo‐inactive in its native state, and the earlier attempts to make it photoactive remained either unsuccessful or required the incorporation of water molecules in the structure, which may not be considered a viable design element. Taking the clue from our earlier studies and using the LSAM concept, a bicomponent salt of BZA and 2NVP was designed, which turned out to be photoreactive. The second key aspect of the study relies on the photomechanical effects associated with the photodimerization in this bicomponent solid. The system shows crystal cracking under UV and visible light, making it one of a few organic systems undergoing the same. Thirdly, this system provides a unique opportunity where crystal cracking due to [2+2] photodimerization in an organic crystal could be monitored through several intermediate structures as it undergoes SC‐SC transformation. This makes it a rare example where photoinduced cracking is slow enough to maintain enough single crystallinity during the [2+2] photoreaction and where photo‐induced cracking could be monitored through structural intermediates. The analysis of crystal packing of these intermediate stages reveals the structural changes associated with the crystal cracking. In the end, this example relates variegated photo responses to large synthons and shows that an LSAM‐based crystal design can eventually lead to property engineering, a direction that crystal engineering has always strived.

## Author Contributions

**MRA**: Data Curation, Formal Analysis, Methodology, Validation, Visualization, Software, Writing‐Original draft preparation; **MKM**: Data Curation (Single Crystal), Validation (Single Crystal); **AM**: Conceptualization, Investigation, Project administration, Funding acquisition, Writing – Review & Editing, Supervision.

## Conflict of Interests

The authors declare no conflict of interest.

1

## Supporting information

As a service to our authors and readers, this journal provides supporting information supplied by the authors. Such materials are peer reviewed and may be re‐organized for online delivery, but are not copy‐edited or typeset. Technical support issues arising from supporting information (other than missing files) should be addressed to the authors.

Supporting Information

## Data Availability

The data that support the findings of this study are available in the supplementary material of this article.
