# Supplementary material for: Photo‐Induced Cracking in a Bi‐Component Molecular Solid: Capturing Structural Intermediates
Source: Chem Asian J. 2025 Feb 26;20(9):e202401518. doi: 10.1002/asia.202401518 (PMC12067860; doi:10.1002/asia.202401518)
Supplement: Supplementary file 1 — Supporting Information [file ASIA-20-e202401518-s001.pdf]

Chemistry - An Asian Journal

Supporting Information

Photo-Induced Cracking in a Bi-Component Molecular Solid: Capturing Structural Intermediates

Mollah Rohan Ahsan, Manish Kumar Mishra, and Arijit Mukherjee*

Photo-induced Cracking in a Bi-component Molecular Solid: Capturing Structural Intermediates

Mollah Rohan Ahsan,[a] Manish Kumar Mishra,[b, c] and Arijit Mukherjee *[a]

[a] Department: Department of Chemistry, BITS Pilani Hyderabad Campus
Shamirpet, Jawhar Nagar, PIN: 500078
E-mail: arijit.mukherjee@hyderabad.bits-pilani.ac.in

[b] Department: Physical and Materials Chemistry Division, CSIR-National Chemical Laboratory,
Homi Bhabha Road, Pashan, Pune, India

[c] Academy of Scientific and Innovative Research (AcSIR),
Ghaziabad 201002, India

Table of Contents

**Sl. No.**	**Content**	**Page No.**
S1.	Material and Methods	1
S2.	Preparation of binary solids	2-3
S3.	Experimental techniques	3-5
S4.	Single Crystal X-ray diffraction (SC-XRD)	5-7
S5.	Analysis of single crystals	8-12
S6.	Structural Overlap	11-12
S7.	Powder X-ray Diffraction (PXRD)	12-13
S8.	Differential Thermal Calorimetry (DSC)	13-15
S9.	Nuclear Magnetic Resonance (NMR)	15-18
S10.	Mass Spectra	18-19
S11.	Check CIF validation	20
S12.	ORTEP Diagrams	21

S1. Materials and Methods:

A. Synthesis: Benzilic acid (BZA) and (E)-4-(2-(naphthalene-2-yl) vinyl) pyridine (2NVP) were prepared using a reported method.[1] BZA was prepared through benzil-benzilic acid rearrangement from benzil using KOH. 2NVP was prepared through the condensation reaction of 4-methylpyridine and 2-naphthaldehyde. 0.94 g of 4-methyl pyridine (10 mmol) and 1.56g of 2-naphthaldehyde (10 mmol) were taken in a round bottle flask with 3 mL acetic acid. The reaction mixture was stirred at 140º C for 48 hours. The crude mixture obtained was dissolved in DCM and worked up using a saturated $NaHCO_3$ solution to neutralize excess acetic acid. The organic layer was separated and dried under a vacuum using a rota-evaporator. The crude product was purified using an EtOAc/hexane mixture through column chromatography. The purity of the synthesized compound was confirmed through ^{1}H NMR and used for further studies. (*E*)-4-(3,5-dichlorostyryl) pyridine (DCP) was also prepared using the mentioned procedure. 1.75g of 3,5-dichlorobenzaldehyde and 0.94 g of 4-methylpyridine were taken in a round bottom flask with 3mL of acetic acid and refluxed at 140 ºC for 40 hours. The formation of the product was confirmed through TLC (20% EtOAc/hexane mixture). The product was purified using column chromatography and dried using a rota-evaporator. The formation of the product was confirmed through ^{1}H NMR and mass spectra.

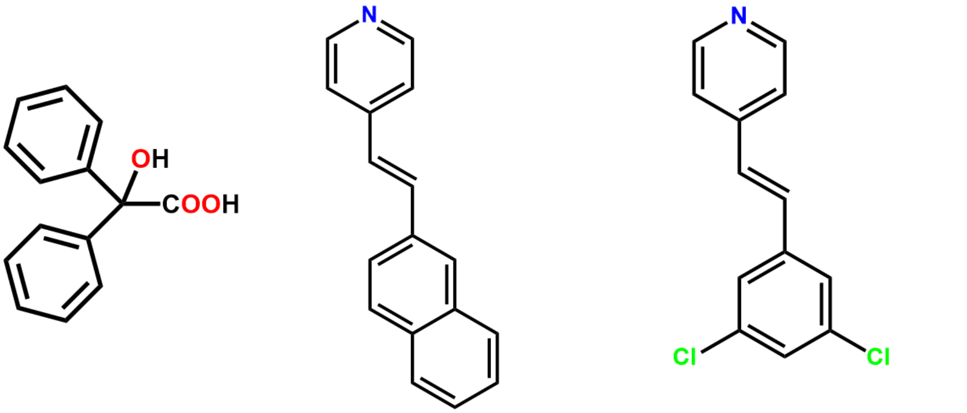

Benzilic acid (BZA) **(E)-4-(2-(naphthalen-2-yl)vinyl)pyridine (2NVP)** **(E)-4-(3,5-dichlorostyryl) pyridine (DCP)**

S2. Preparation of the binary solids:

(a) BZA-2NVP: The binary salts were prepared using liquid-assisted grinding and solution crystallization. The two coformers, BZA (45.2mg) and 2NVP (46.4mg), were ground using 3-4 drops of acetonitrile for 12-15 minutes. The ground mixture was dissolved in several organic solvents such as acetonitrile, ethanol, 1,4-dioxane, and DMF. Yellow block-shaped crystals were obtained in all these cases. Solution crystallization from acetonitrile and DCM also gave similar crystals.

(b) Crystallization from ionic liquids: Apart from using typical organic solvents, we employed ionic liquids as a crystallization solvent.[2] Around 10 mg of the ground mixture of BZA and 2NVP were taken in a vial and dissolved in two different ionic liquids such as 1-Ethyl-3-methylimidazolium bis (trifluoromethylsulfonyl) imide ($[Emim]^+ [NTF_2]^-$) (10 µl) and 1-(2'-hydroxyethyl)-3-methylimidazolium bis(trifluoromethylsulfonyl) imide ($[OH\text{-}Emim]^+[NTF_2]^-$) (10 µl) through heating. Subsequent cooling crystallization generated the yellow-colored block-shaped crystals.

(c) BZA-DCP Form II: The Form II of the BZA-DCP was obtained through solution crystallization. 11.6 mg of the benzilic acid and 12.5 mg DCP were weighed, dissolved in ~3 mL of ethanol and acetonitrile solution, and kept for slow evaporation. The colorless block-shaped crystals obtained after 3-4 days were used for further characterization.

S3. Experimental section:

(a) Single Crystal XRD: The crystals of BZA-2NVP were exposed directly to sunlight for photochemical reactions. For single-crystal X-ray diffraction (SCXRD) analysis, crystals were selected at different intervals of sunlight exposure: ***I, II, III, and IV***. The SCXRD studies of the crystal were performed using a Bruker SMART APEX II single-crystal X-ray CCD diffractometer with graphite-monochromatized (Mo-K= 0.71073 Å) radiation at 100 K. The unit cells were determined using a total of three matrix runs comprising a total of 36 frames (images). The complete intensity data were collected by using an optimized strategy with different sets of ω, φ, and 2θ and with 0.5° width, keeping the sample-to-detector distance fixed at 5.00 cm. The X-ray generator was maintained at 50 kV and 30 mA, and the data were collected within 10-20 s. The X-ray data acquisition was controlled and monitored by the APEX2 program suite (Bruker, 2006). The complete data sets were corrected for Lorentz-polarization and absorption effects using the APEX3 package (Bruker, 2016) through the SAINT and SADABS programs.

All four crystal structures were solved using SHELXT (Intrinsic Phasing) methods.[3] Hydrogen atoms or protons bonded to the nitrogen atom of the **2NVP**H^+ cation were located from the difference Fourier map. Hydrogen atoms bonded to other atoms were added per the calculated values using a riding model during refinement. All non-hydrogen atoms were refined using anisotropic displacement parameters. The two carbon atoms (C6 and C7) involved in photoactive double bonds of **2NVPH⁺** cation of **II and III crystals** have positional disorders. The disorder has been refined using Part 1 and Part 2 instructions. The disordered carbon atoms C24A and C25A positions in **II and III** were located from the difference map. The site occupancies (%) for both the ordered carbon atoms (C24B and C25B) and the carbon atoms (C24A and C25A) are provided in Table 1 below. The disorder refinement strategy is available in the deposited CIF files.

Table 1. Percentage occupancies (%) of positional of ordered (C24A and C25A) and (C24A and C25B) carbon atoms in the crystals.

Crystal	Ordered C24B and C25B atoms occupancies (%)	Disordered C24A and C25A atoms occupancies (%)
**I**	100	0
**II**	~80	~20
**III**	~45	~55
**IV**	20	~80

(b) Powder X-ray Diffraction (PXRD): PXRD for the native and binary solids was collected using a Rigaku X-ray diffractometer. The PXRD data was collected in a range of 5-40° with a scan rate of 2°/min and a step size of 0.01. The simulated patterns were generated using Mercury software[4] from the final refined CIF file. A stacked plot between simulated and experimental patterns was plotted using X'Pert High Score Plus software.[5]

(c) Differential Thermal Calorimetry (DSC): DSC data for native and binary solid samples were collected using the Shimadzu DSC instrument within 30-250ºC, with a ramp rate of 5 ºC/min.

(d) Nuclear Magnetic Resonance (NMR): NMR data of the synthesized compound and binary solids (before and after irradiation) were collected using a Bruker 400MHz instrument. The NMR data were collected by dissolving ~5 mg sample in $CDCl_3$.

(e) Mass Spectra: Mass spectra of the irradiated and native samples were collected by dissolving a pinch of sample in 1.5 mL of methanol; the data were collected using the Shimadzu LCMS-4000 instrument.

(f) Photo Irradiation: Photoirradiation was performed under broadband UV radiation and visible light. Single crystals of the binary solids were kept over Pyrex glass slides and irradiated for various intervals. The images and other characterizations were performed using these irradiated crystals.

(g) Microscope: An OLYMPUS BX53M microscope was used to collect microscopic images.

(h) Confocal Microscope: Confocal images of respective samples were collected using the Leica TCS SP8 instrument. The single crystals were excited at a wavelength of 420nm with an 8% lesser power.

(i) Field emission scanning electron microscopy (FE-SEM): A FEI Apreo LoVac FE-SEM instrument was used to visualize the cracks generated due to photoirradiation.

(j) Computational Calculations: An ESP calculation was performed for DCP, 1NVP, and 2NVP using Orca[6] and Multiwfn software[7] and visualized through VMD.[8] A def2-TZVP basis set was used to optimize the structures, and the ESP was generated from the optimized structure.

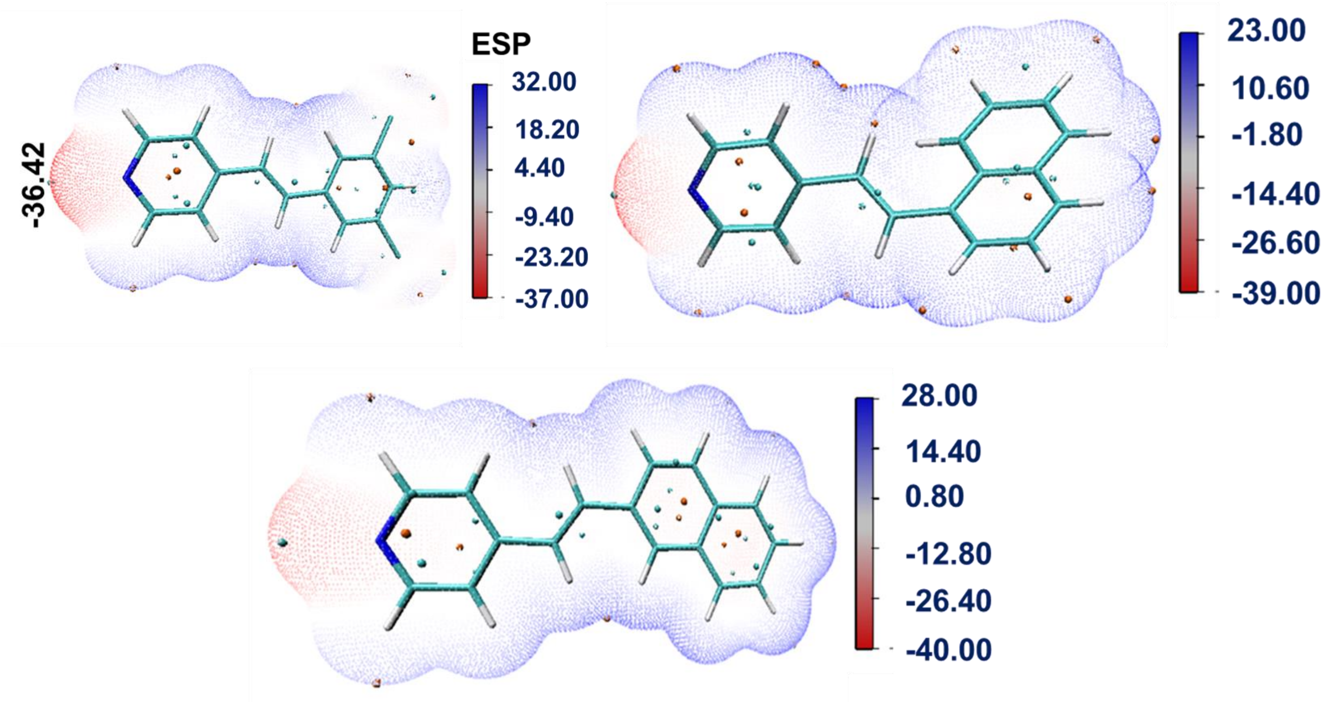

S4. Single Crystal X-Ray Diffraction:

(b) Crystallographic Table:

compound	**I**	**II**	**III**
Empirical formula	$C_{31} H_{25} NO_3$	$C_{62} H_{50} N_2 O_6$	$C_{62} H_{50} N_2 O_6$
CCDC NO.	2393974	2393975	2393976
Formula Weight	459.52	919.04	919.04
Crystal System	$P2_1/n$	$P2_1/n$	$P2_1/n$
Space Group	*Monoclinic*	*Monoclinic*	*Monoclinic*
a (Å)	8.6372(8)	8.6453(16)	8.7894(18)
b (Å)	16.4566(12)	16.398(3)	16.182(4)

c (Å)	16.8871(13)	17.001(3)	17.390(4)
α (˚)	90	90	90
β (˚)	103.164(3)	102.967(6)	101.561(5)
γ (˚)	90	90	90
V (Å3)	2337.2(3)	2348.7(7)	2423.2(10)
ρ_{calc} (g/cm3)	1.306	1.299	1.260
F (000)	968.0	968.0	968.0
μ. (mm-1)	0.084	0.083	0.081
T (K)	107	111	100
λ (Å)	0.71073	0.71073	0.71073
Total Reflections.	4920	3098	2965
Unique Reflection	4422	2503	1821
Completeness (%)	99.3	99.9	99.8
R_{int}	5.66	8.49	11.77
R_1 (F^2)	6.91	7.24	7.29
wR_2(F^2)	16.93	17.24	18.06
GoF	1.102	1.131	1.077
Θ_{max}	26.731	22.575	21.990
compound	**IV**	**BZA-DCP-Form II**	----------------
Empirical formula	$C_{62}H_{50}N_2O_6$	$C_{27}H_{21}NO_3Cl_2$	----------------
CCDC NO.	2393977	2393978	----------------
Formula Weight	919.04	478.35	----------------
Crystal System	$P2_1/n$	$P\overline{1}$	----------------
Space Group	*Monoclinic*	Triclinic	----------------
a (Å)	8.642(7)	10.0005(5)	----------------
b (Å)	15.920(13)	10.8484(6)	----------------
c (Å)	17.265(12)	12.2088(6)	----------------
α (˚)	90	73.312(2)	----------------
β (˚)	101.907(18)	71.064(2)	----------------
γ (˚)	90	69.036(2)	----------------

V (Å3)	2324(3)	1147.77(10)	----------------
ρ_{calc} (g/cm3)	1.313	1.384	----------------
F (000)	968.0	496.0	----------------
μ. (mm-1)	0.084	0.313	----------------
T (K)	100	100	----------------
λ (Å)	0.71073	0.71073	----------------
Total Reflections.	2744	5921	----------------
Unique Reflection	1827	4907	----------------
Completeness (%)	99.9	99.4	----------------
R_{int}	16.85	8.0	----------------
R_1 (F^2)	8.23	5.10	----------------
wR_2(F^2)	20.91	13.65	----------------
GoF	1.065	1.041	----------------
Θ_{max}	21.738	28.751	----------------

(b) Hydrogen Bonding Table:

**structure**	**interaction**	**D-H (Å)**	**H···A (Å)**	**D···A (Å)**	**<(DHA) (°)**
**I**	N4-H4···O1	0.95(4)	1.59(4)	2.547(2)	179(4)
	O3-H3···O2	0.83(4)	1.96(3)	2.573(2)	131(3)
**II**	N4-H4···O1	1.24(5)	1.31(5)	2.548(4)	178(4)
	O3-H3···O2	0.88(6)	1.88(6)	2.574(4)	134(5)
**III**	N4-H4···O1	1.20(7)	1.34(7)	2.534(5)	174(5)
	O3-H3···O2	0.82	2.02	2.544(5)	120.8
**IV**	N4-H4···O1	1.02(8)	1.51(8)	2.527(6)	177(6)
	O3-H3···O2	0.79(8)	1.98(8)	2.540(7)	129(8)
**BZA-DCP-Form II- Before Irradiation**	N8-H8···O5	1.01(3)	1.53(3)	2.5426(17)	178(3)
	O7-H7···O5	0.86(3)	1.92(3)	2.5406(17)	128(3)

S5. Analysis of single crystals:

(a) Face indexing of zero hour crystal:

Face indexing of a well-shaped crystal at zero hour was done by Bruker D8 Venture, Kappa Duo PHOTON II CPAD diffractometer with a Mo micro-focus sealed X-ray tube [λ (Mo Kα = 0.71073 Å, generator power 50 kV and 1.4 mA] and InfoTech multilayer mirror optics. We identified one prominent face as (01-1)/(0-11), along with several minor faces.

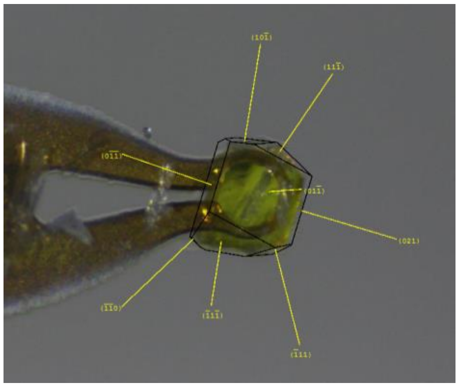

(b) Face indexing of 0h crystal through BFDH calculation: The final refined structure of **I** was used to generate the morphology. CSD-Particle embedded in Mercury 4.2.0[4] was used to create the BFDH morphology of the crystal.

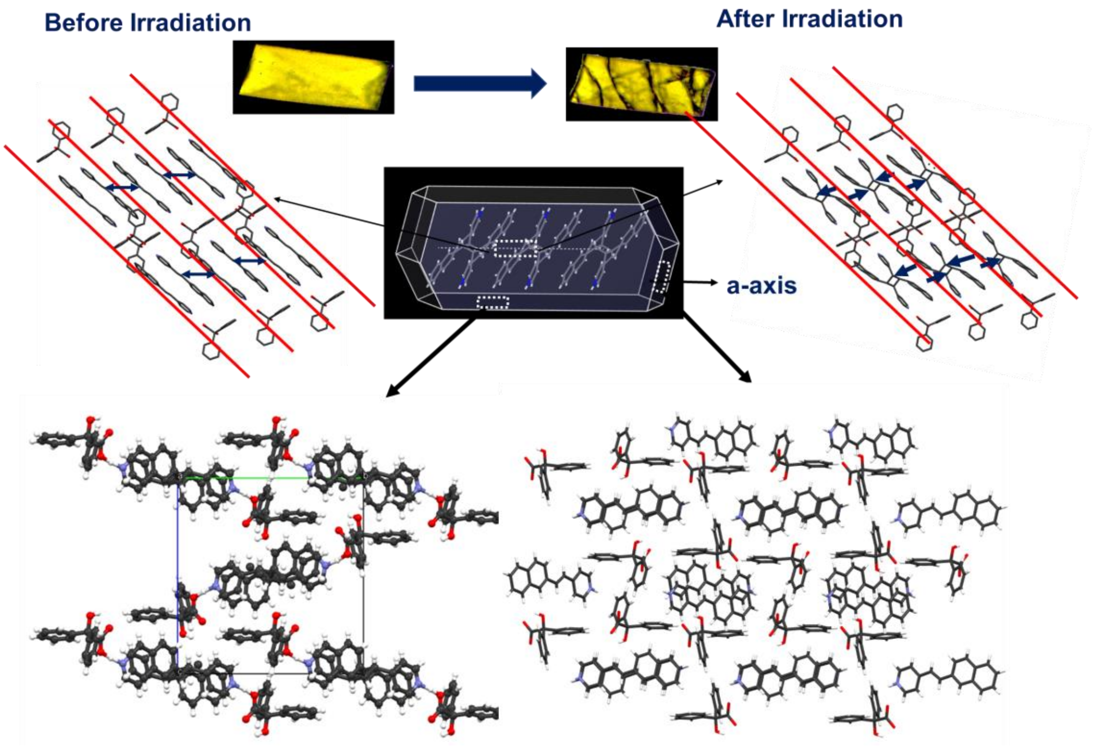

(c) Crystal images at different sunlight exposures:

<table>
<tr><td>I</td><td>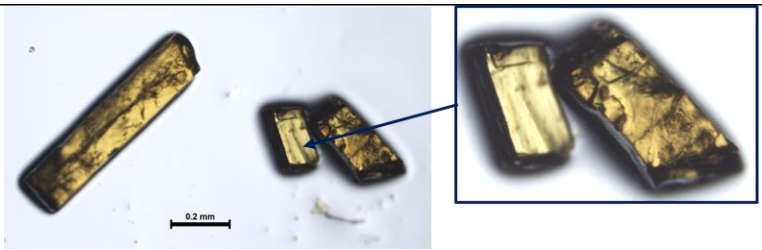
</td></tr>
<tr><td>II</td><td>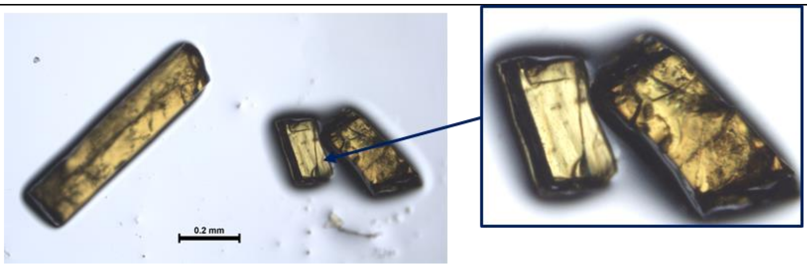
</td></tr>
<tr><td>III</td><td>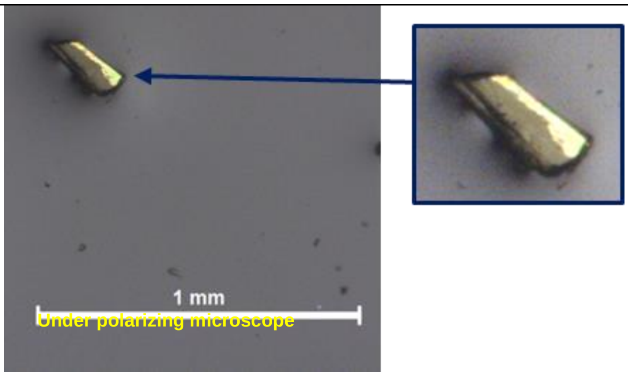
</td></tr>
<tr><td>IV</td><td>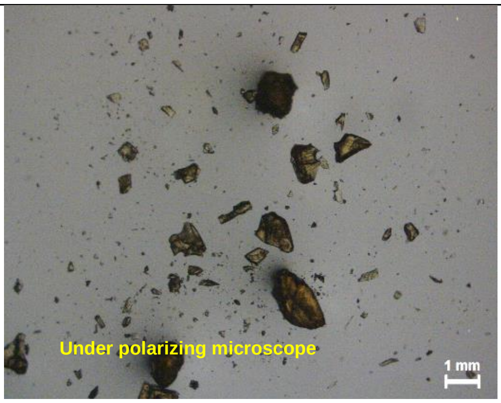
</td></tr>
</table>

(d) Average Crack distribution: Multiple single crystals were irradiated, and the cracks generated were visualized under a microscope. The distance between the cracks and the angle of the cracks were measured using a microscope. The distance between the cracks varied between 47-75 µm with an average separation of 62 µm and an average crack angle of 60°.

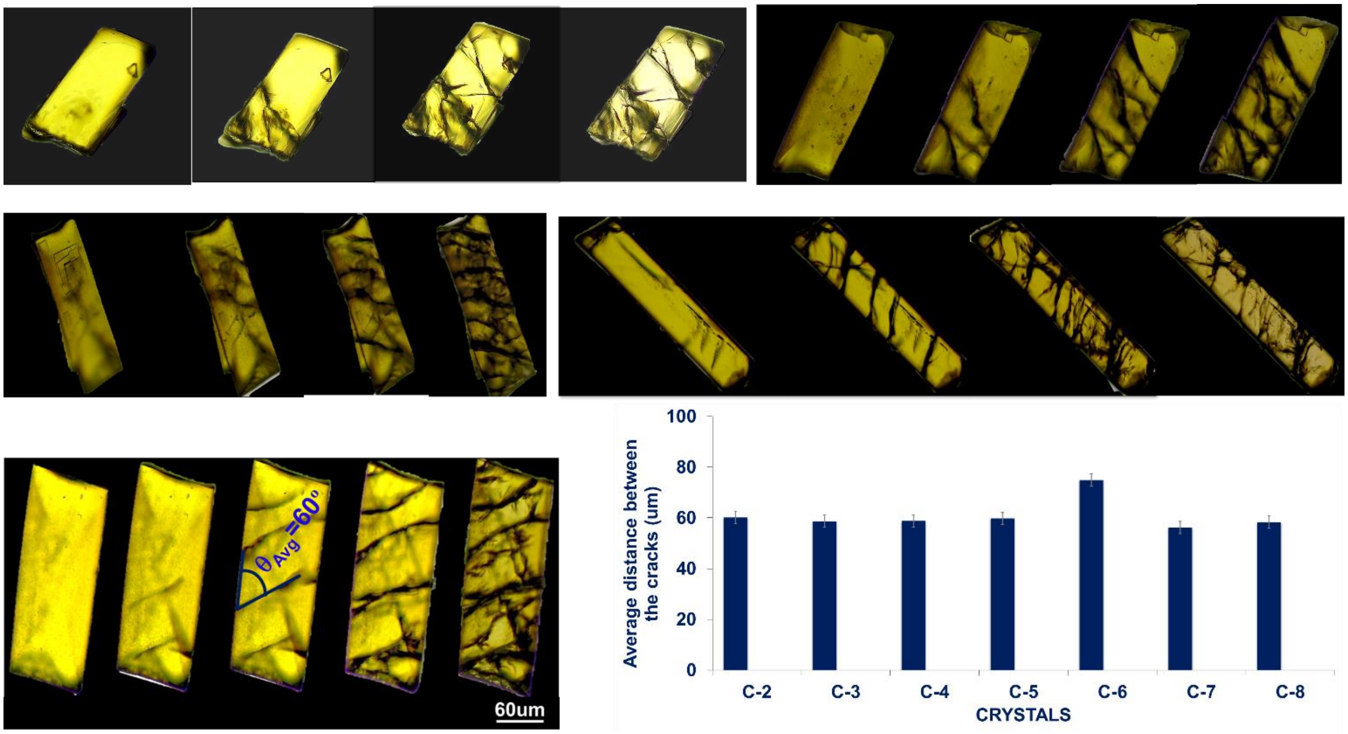

(e) Calculation of parallel layers in cracked crystals: Average crack distance is 62 μm. The distance between the parallel layers is 3.62 Å. So, the number of parallel lyers that likely recated to accumulate the strain is $(62 * 10^4)/3.62 \approx 17*10^4$.

(f) SEM Images of the cracked and disintegrated crystals:

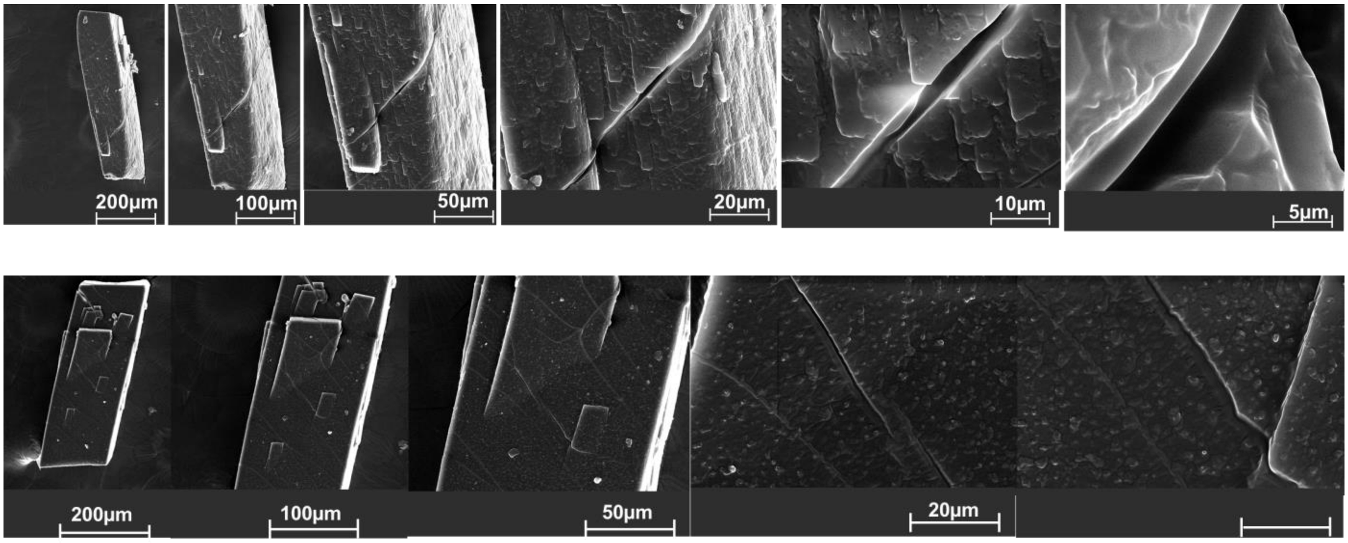

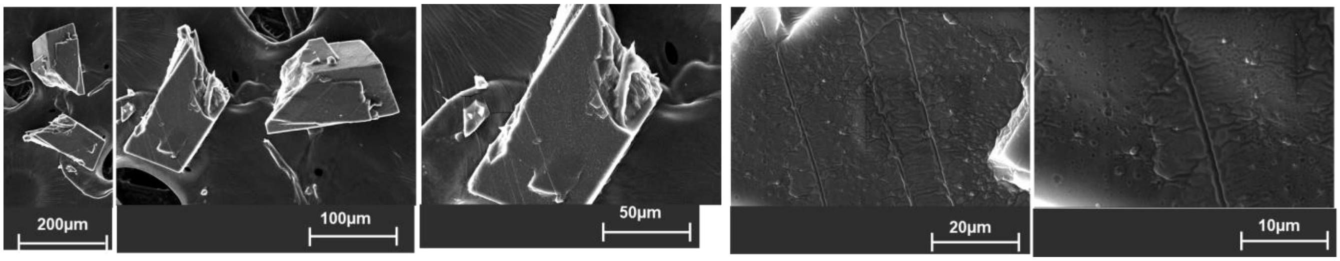

S6. Structural Overlap: The structures collected at different time intervals of irradiation were overlapped for comparison, using Mercury 4.2.0 software.

(i) I and II:

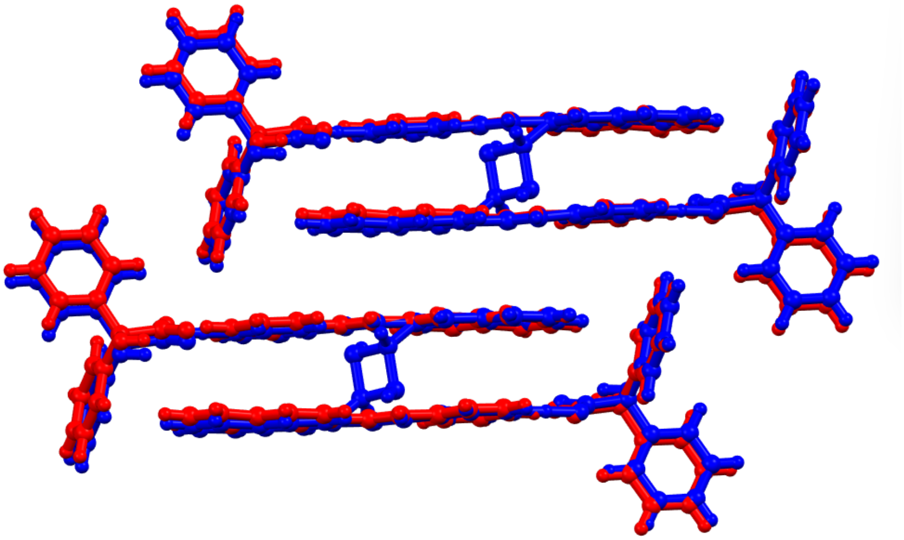

(ii) I and III:

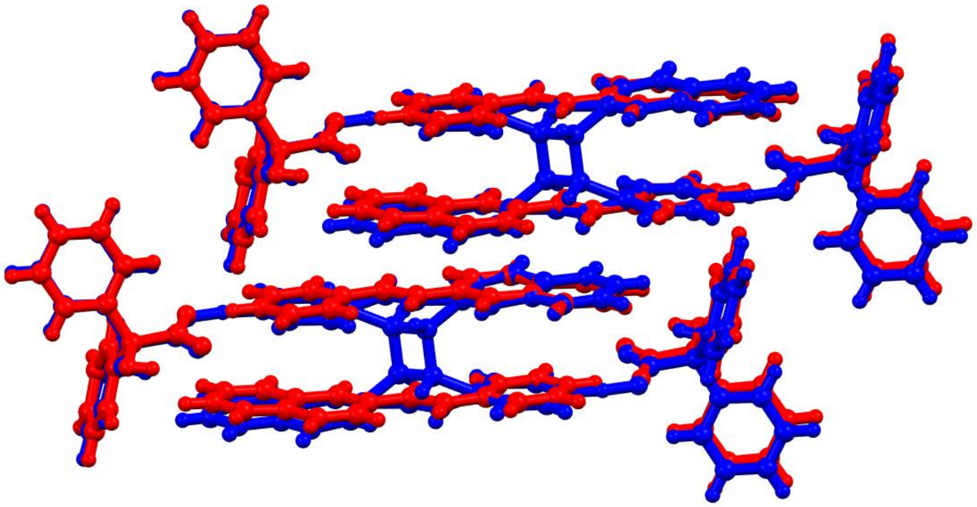

(iii) I and IV:

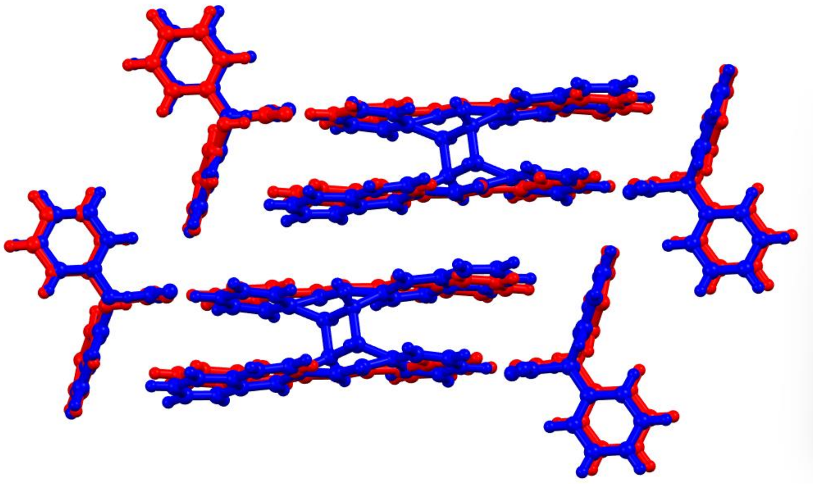

S7. Powder X-Ray Diffraction (PXRD):

(i) PXRD data of I: The sample for the PXRD was prepared as mentioned in the ESI-S2. All the experimental peaks were matched with the simulated pattern, confirming the formation of the binary solid. The absence of any peaks other than the simulated pattern confirmed the phase purity of the material.

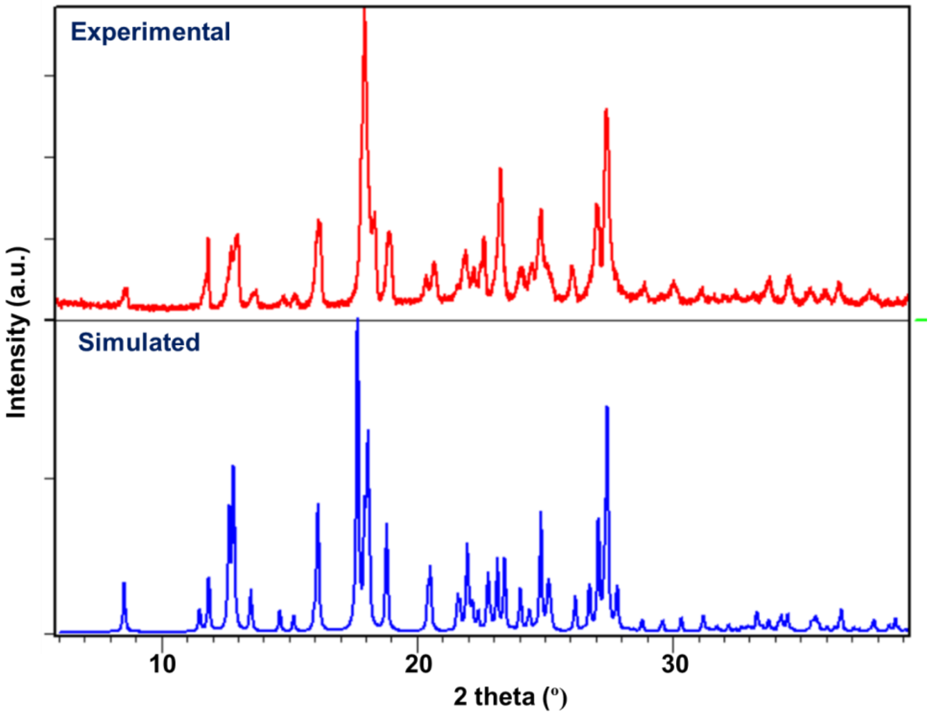

(ii) PXRD data of I crystals: Crystals obtained from the solution were used as such (without grinding), and PXRD data was collected to obtain the major faces.

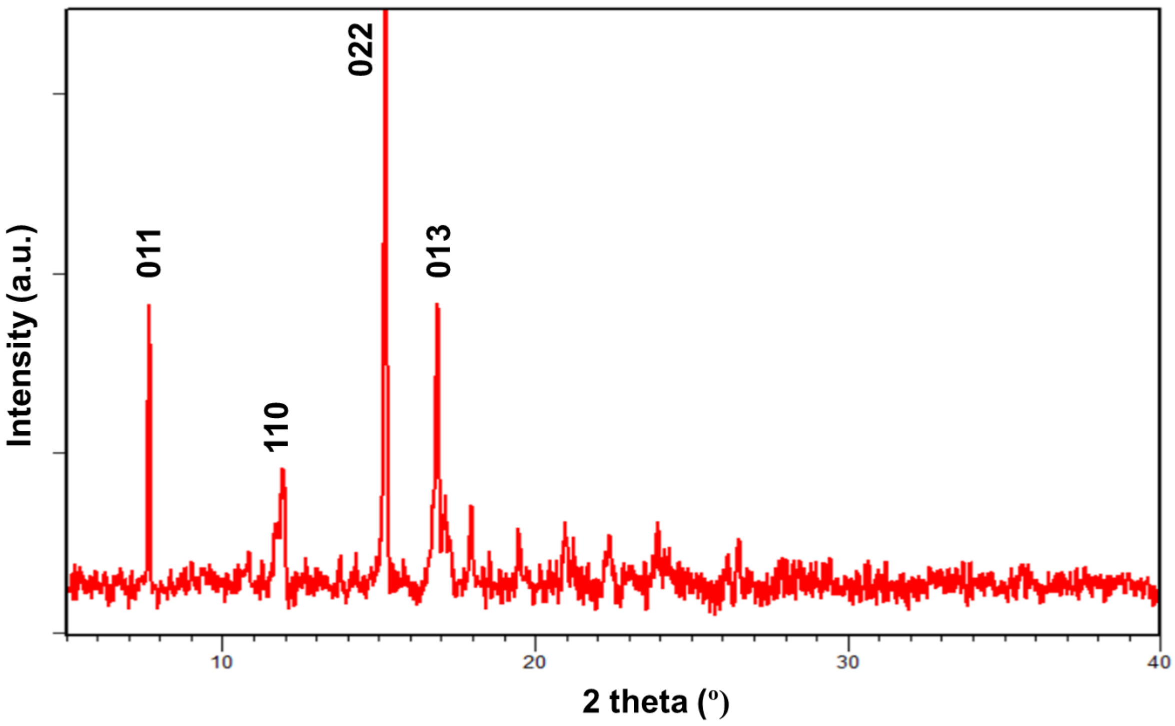

(iii) PXRD data of BZA-DCP Form II: The BZA-DCP Form II was obtained only through solution crystallization from ACN and ethanol. The crystals obtained from the solution were ground gently using mortar and pestle, and PXRD data of the ground sample was collected.

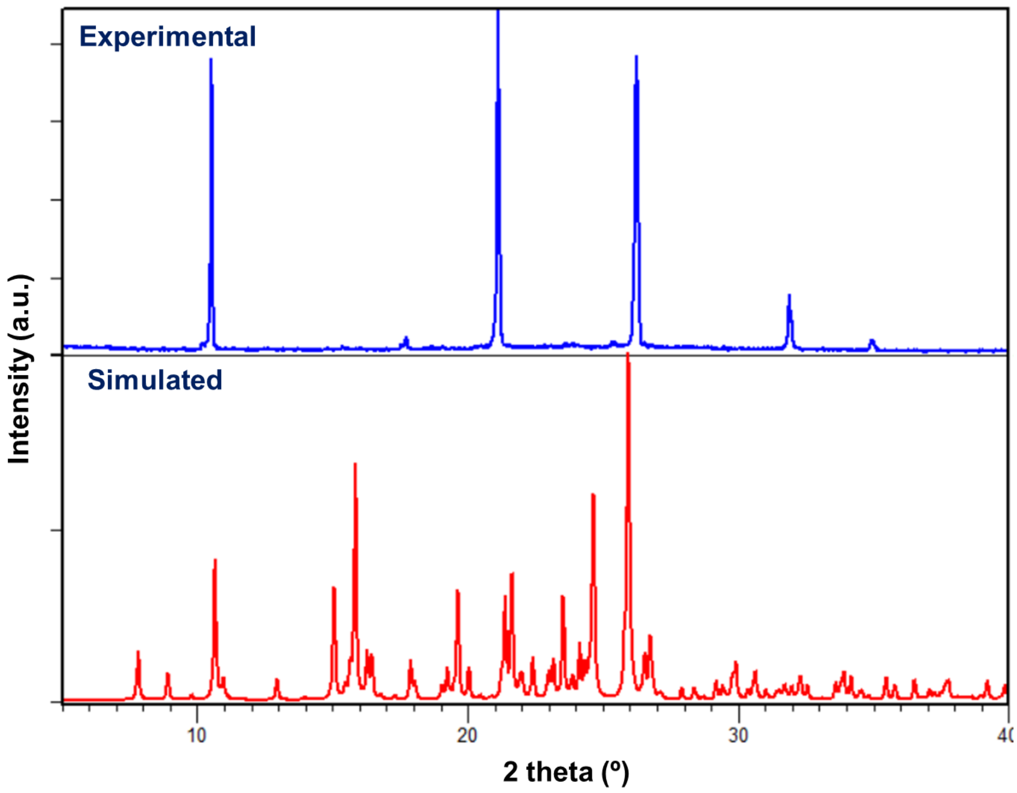

S8. Differential Scanning Calorimetry (DSC):

(a) DSC data of 2NVP: The DSC data of the purified 2NVP was collected using an aluminum pan in an N_2 atmosphere, within a range between 30-250ºC. The onset appeared at 157.42ºC with a melting enthalpy of -96.22 J/g.

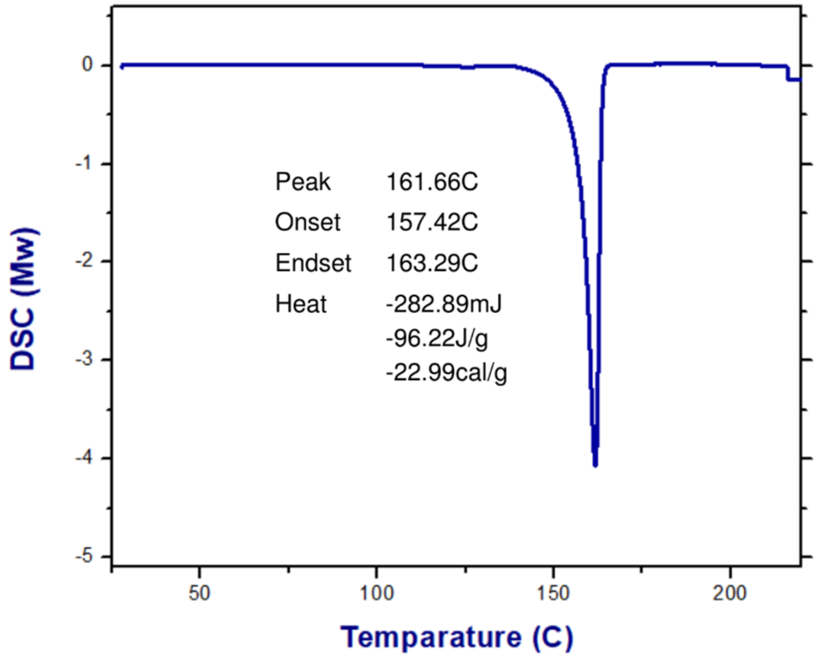

(b) DSC data BZA-2NVP: The crystals obtained were ground using mortar and pestle, and DSC data was collected for the sample. The melting onset appeared at 162.01ºC with a melting enthalpy of -44.01 J/g.

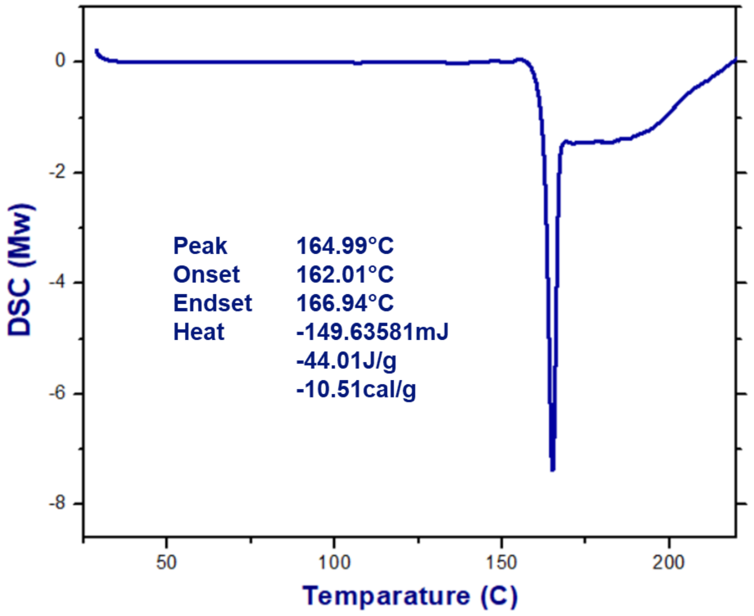

(c) DSC data BZA-DCP-Form II: The crystals obtained from solution crystallization from ACN were ground using mortar and pestle, and DSC data was collected for the sample. The melting onset appeared at 173.14ºC with a melting endotherm of -68.75 J/g.

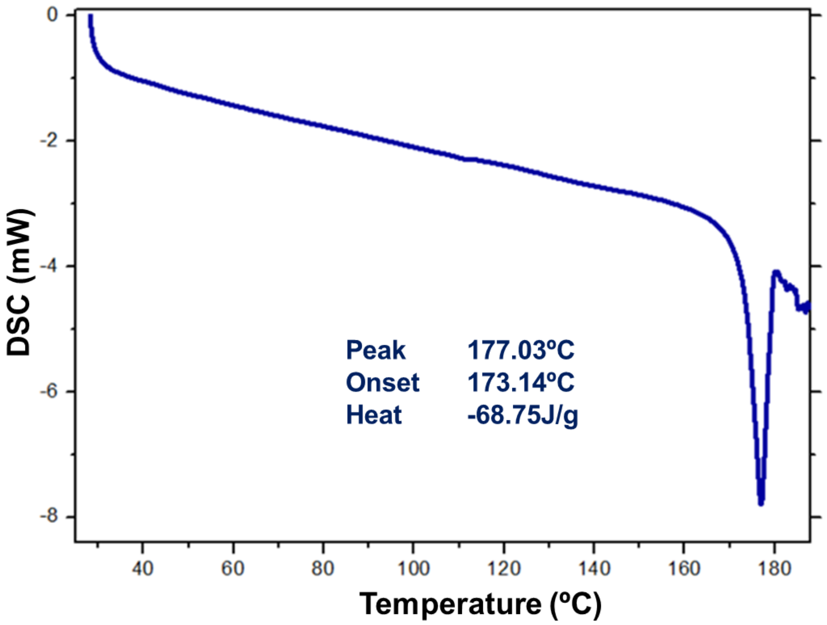

S9. Nuclear Magnetic Resonance (NMR):

(a) ^{1}H NMR data of purified (E)-4-(2-(naphthalen-2-yl)vinyl) pyridine (2NVP): The NMR data of the purified 2NVP were collected by dissolving ~5 mg sample in $CDCl_3$. The H_a and H_b protons appeared for the ortho and meta Hs of the pyridyl ring. The H_c peak with a coupling constant of 16.2 Hz confirms the presence of an anti-product. The integration of the H_c proton appeared as 1 when H_a or H_b were integrated as 2. The absence of any other peaks confirmed the purity of the product.

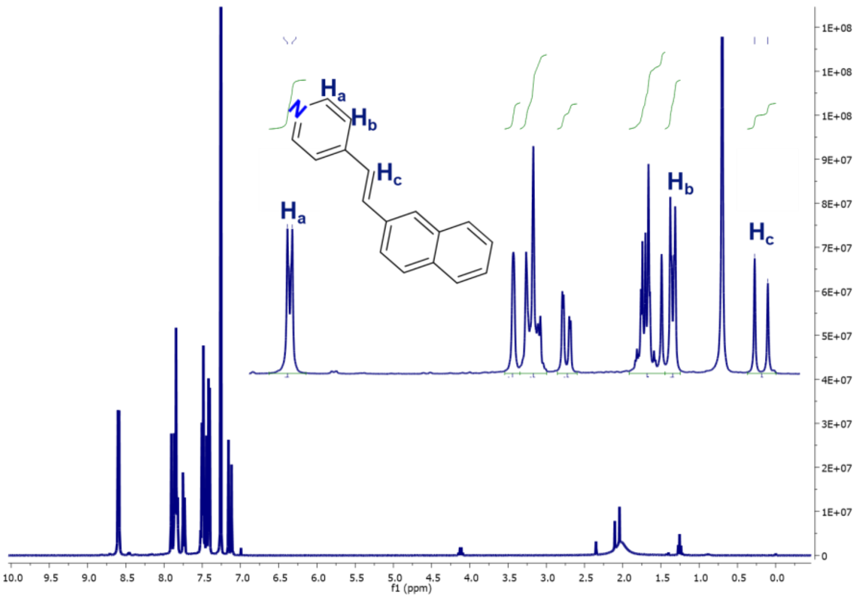

(b) ^{1}H NMR data of BZA-2NVP before and after irradiation: The NMR data were collected by dissolving around 5 mg (before and after irradiation) sample in $CDCl_3$. The appearance of the new peaks at 4.6-4.7 ppm confirmed the formation of the cyclobutane ring. The peak at 8.6 ppm of the before-irradiated sample was shifted to 8.34 ppm; the complete shift of the 8.6 ppm peak confirmed the complete product formation. When the peak at 4.6-4.7 ppm was integrated as 2, the peak at 8.34 ppm also appeared as 2. A similar integration of the 8.34 ppm peak and 4.6-4.7 ppm peak confirmed single product formation during irradiation.

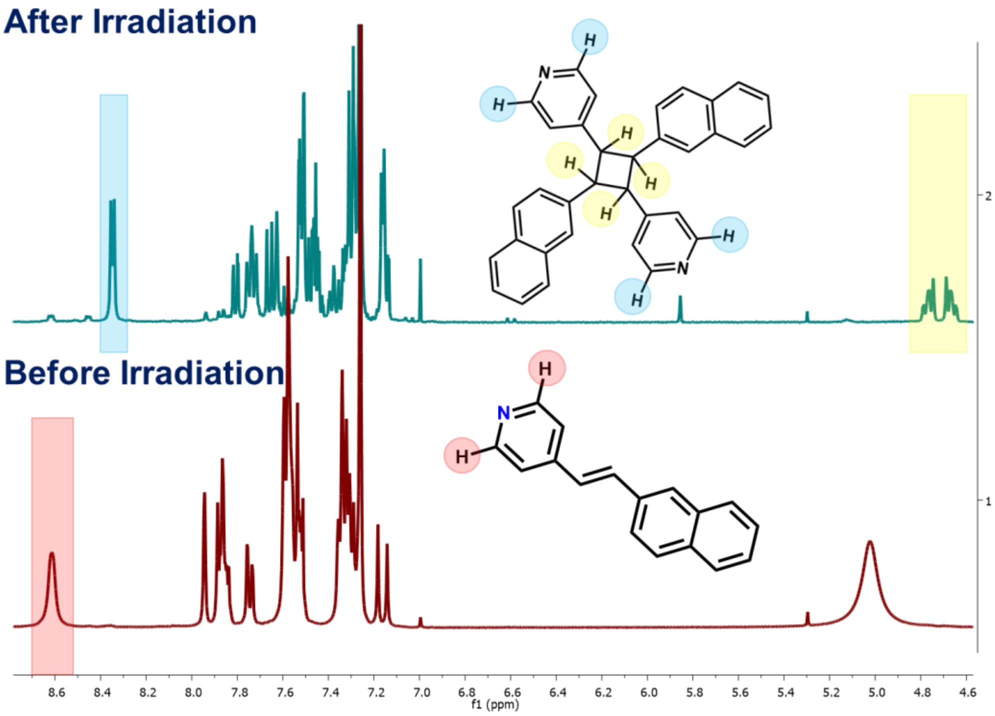

(c) ^{13}C NMR data of BZA-2NVP before and after irradiation: A new peak at 47 ppm appeared for the cyclobutane formation in the ^{13}C NMR.

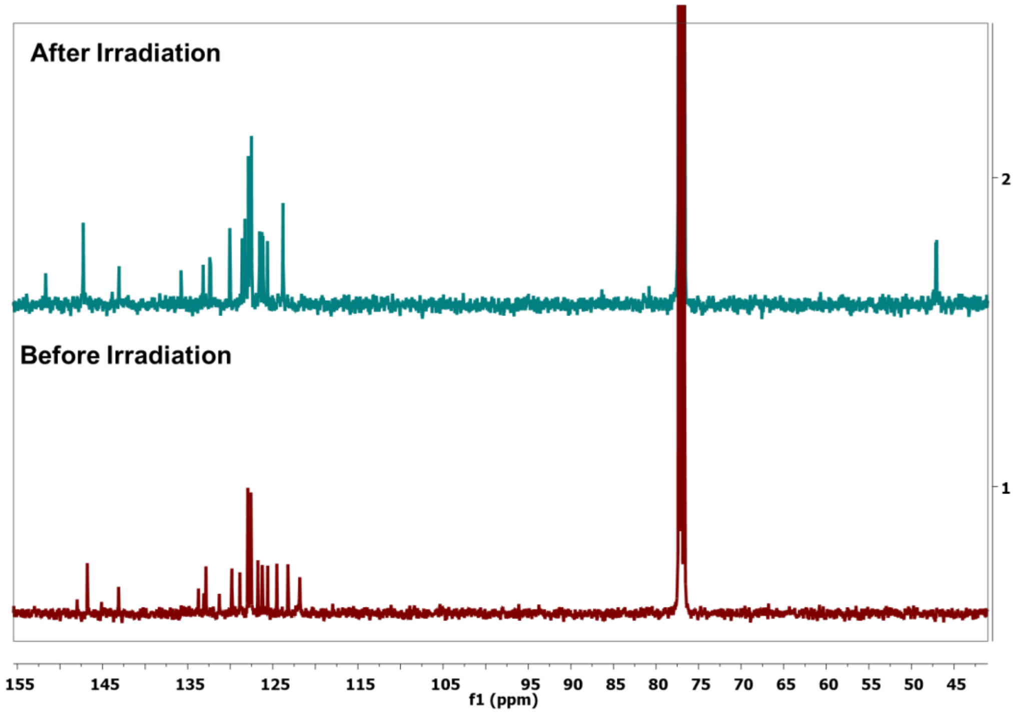

(d) ^{1}H NMR data of BZA-DCP-Form II before and after irradiation: The crystals of BZA-DCP-Form II were taken in a glass slide and irradiated for 10 hours under broadband UV radiations. The irradiated samples were melted after 10 hours; the melted sample of around 7 mg was dissolved in 0.5mL of $CDCl_3$, and the ^{1}H NMR was collected. A new peak appeared around 4.32-4.47 ppm due to the aliphatic protons of the cyclobutane ring; this confirmed the [2+2] cycloaddition reaction. In the unirradiated sample, there was a peak at 8.59 ppm for the pyridine ring's ortho protons (2Hs). After the sample was irradiated under UV radiation, the protons were shifted to 8.47 ppm due to the ortho protons of the pyridine ring of the cyclobutane product. When the peak of 8.47 ppm was integrated as 2, the proton integration of 4.32-4.47 ppm also appeared as 2. The complete shift of the 8.59 peak to 8.47 ppm confirmed the complete conversion to the product, and the similar integration of the ortho proton of the pyridine ring and the cyclobutane proton confirmed the formation of a single product during irradiation.

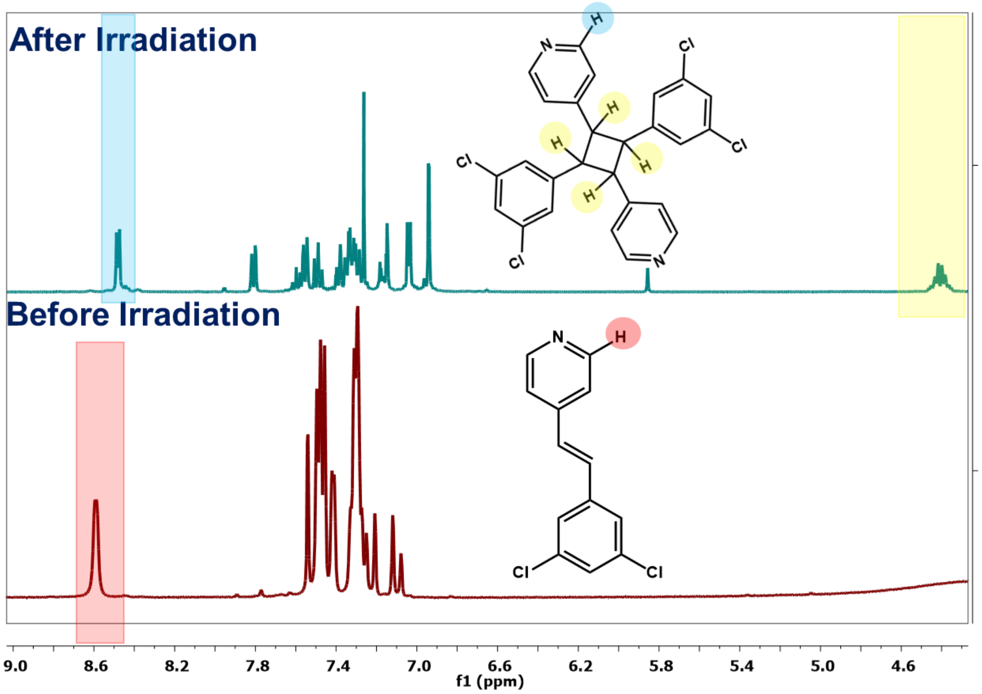

(e) ^{13}C NMR data of BZA-2NVP before and after irradiation: A new peak at 45-47 ppm confirmed the formation of the cyclobutane.

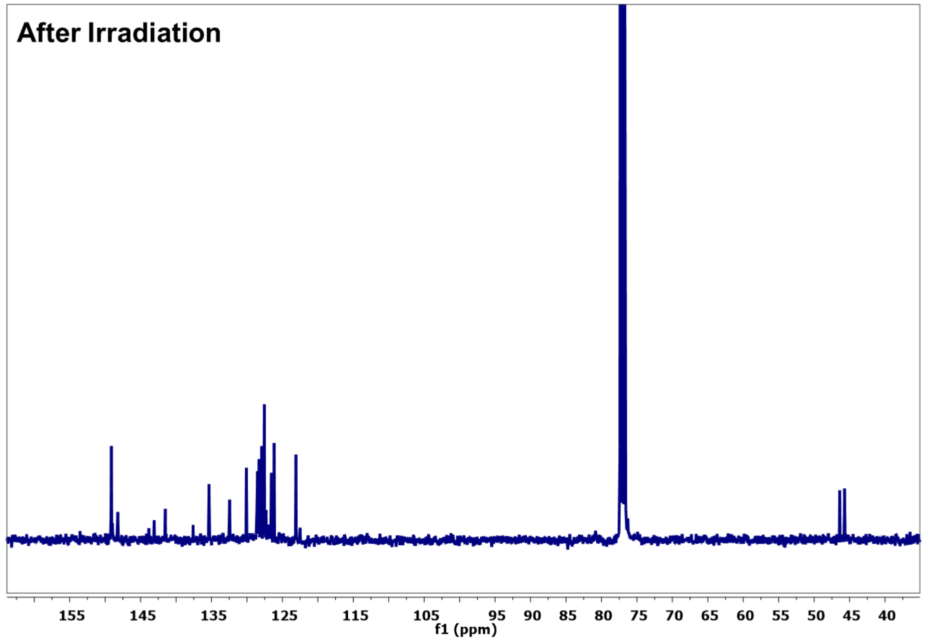

S10. Mass Spectra:

(a) Mass spectra of BZA-2NVP before and after irradiation: In the before-irradiated sample, only one major peak was observed because of the 2NVP molecule's molecular ion peak at m/z=232. In the

irradiated sample, two major peaks appeared, one at m/z=464, because of the molecular ion peak of the 4,4'-(2,4-di(naphthalen-2-yl) cyclobutane-1,3-dial) dipyridine and another major peak was observed at m/z=232 due to the fragmentation of the cyclobutane ring.

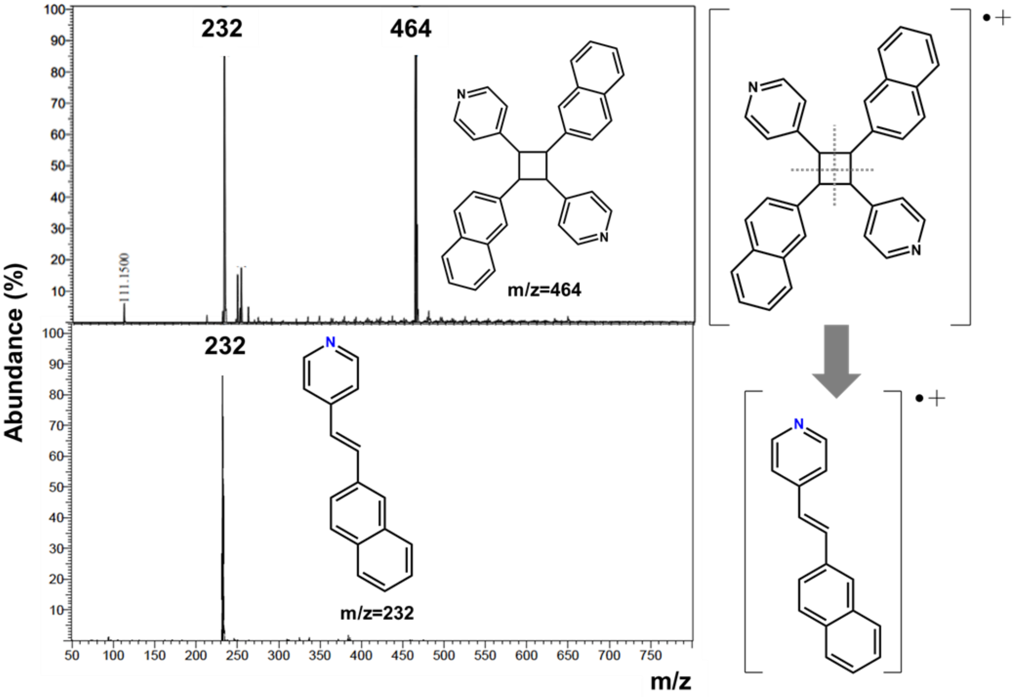

(b) Mass spectra of BZA-DCP-Form II before and after irradiation: In the before-irradiated sample, only one peak was observed at m/z=250. A new peak at m/z=500 was observed due to the dimer.

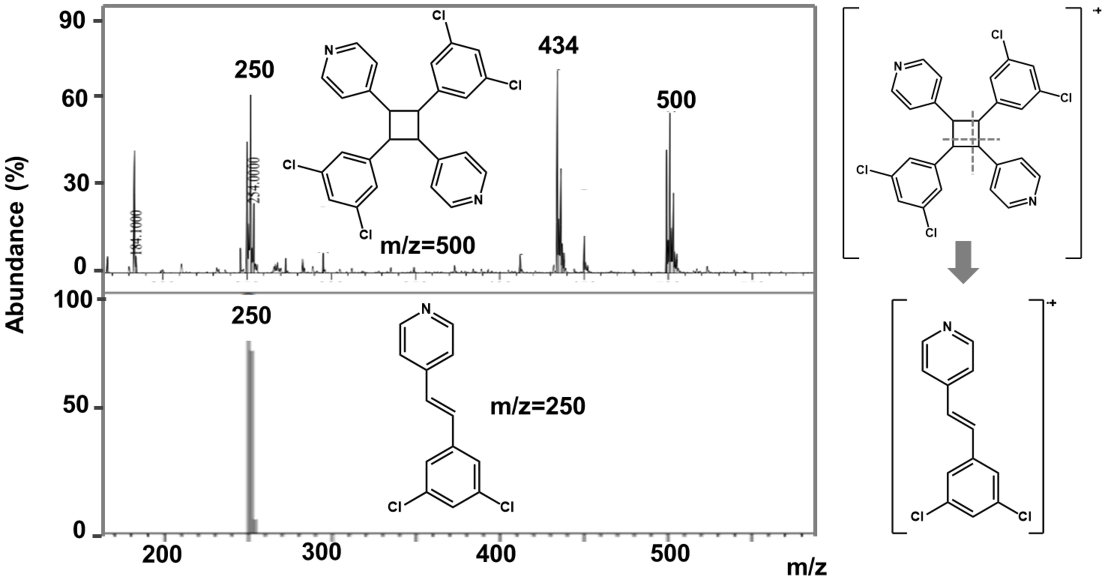

S11. Reply to CheckCIF alerts:

(a) Reply to the alerts for II

(i) *THETM01_ALERT_3_A The value of sine(theta_max)/wavelength is less than 0.550. Calculated sin(theta_max)/wavelength = 0.5401*

Reply: The crystals are reactive to visible light, forming numerous cracks on the crystals that are visible under the microscope. Prolonged exposure to sunlight or visible light degrades the crystal quality. Interestingly, the crystals transition into a glassy or amorphous state as the photoreaction progresses. Although crystal data was collected up to a 0.73 Å resolution at 100 K, the largest available crystal still exhibited weak diffraction at high angles.

(ii) *PLAT353_ALERT_3_A Long N-H (N0.87, N1.01A) N4 - H1. 1.23 Ang.*

Reply: ...Δpk_a calculation results for the system indicate possible salt formation. Therefore, the N-H bond length was higher than the normal bond length, which is why this A alert was generated.

(b) Reply to the alerts for III

(i) *THETM01_ALERT_3_A The value of sine(theta_max)/wavelength is less than 0.550. Calculated sin(theta_max)/wavelength = 0.5268*

Reply: The crystals are reactive to visible light, resulting in the formation of numerous cracks on the crystals that are visible under the microscope. Prolonged exposure to sunlight or visible light degrades the crystal quality. Interestingly, as the photoreaction progresses, the crystals transition into a glassy or amorphous state. Although crystal data was collected up to a 0.73 Å resolution at 100 K, the largest available crystal still exhibited weak diffraction at high angles.

(c) Reply to the alerts for IV

(i) *THETM01_ALERT_3_A The value of sine(theta_max)/wavelength is less than 0.550. Calculated sin(theta_max)/wavelength = 0.5211*

Reply: The crystals are reactive to visible light, resulting in the formation of numerous cracks on the crystals that are visible under the microscope. Prolonged exposure to sunlight or visible light degrades the crystal quality. Interestingly, as the photoreaction progresses, the crystals transition into a glassy or amorphous state. Although crystal data was collected up to a 0.73 Å resolution at 100 K, the largest available crystal still exhibited weak diffraction at high angles.

S12. ORTEP Diagram: ORTEP diagrams were plotted using Mercury 4.2.0 software with 50% probability.

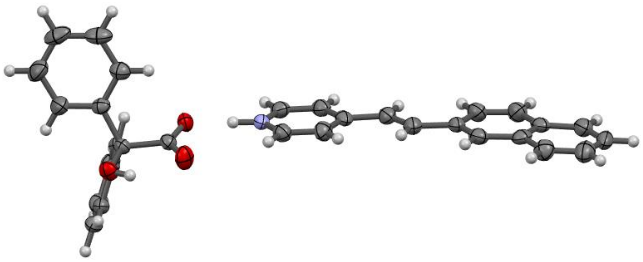	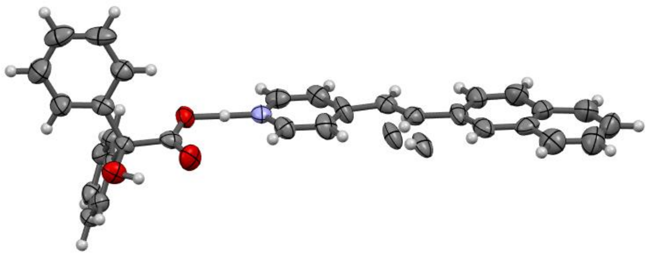
**I**	**II**
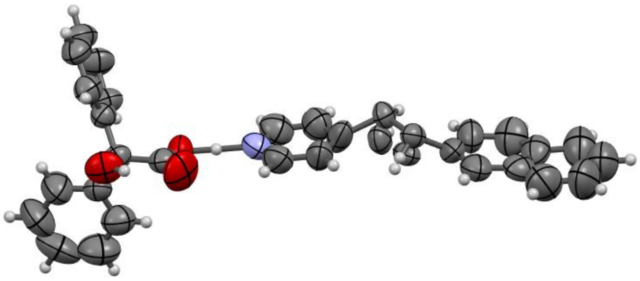	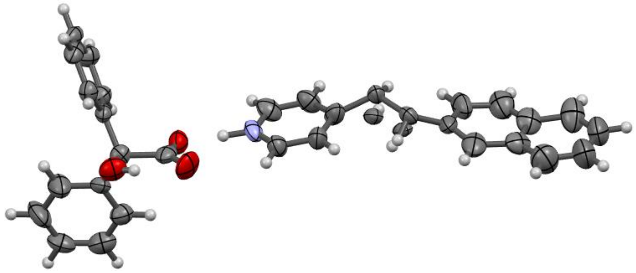
**III**	**IV**
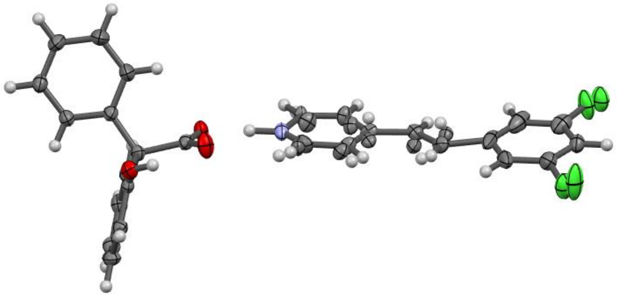	
**BZA-DCP-Form II-Before Irradiation**	

References:

[1] J. L. R. Williams, R. E. Adel, J. M. Carlson, G. A. Reynolds, D. G. Borden, J. A. Ford, *J. Org. Chem.* **1963**, *28*, 387–390.

[2] Q. Zeng, A. Mukherjee, P. Müller, R. D. Rogers, A. S. Myerson, *Chem. Sci.* **2018**, *9*, 1510–1520.

[3] G. M. Sheldrick, *Acta Crystallogr. C Struct. Chem.* **2015**, *71*, 3–8.

[4] C. F. Macrae, I. Sovago, S. J. Cottrell, P. T. A. Galek, P. McCabe, E. Pidcock, M. Platings, G. P. Shields, J. S. Stevens, M. Towler, P. A. Wood, *J. Appl. Crystallogr.* **2020**, *53*, 226–235.

[5] T. Degen, M. Sadki, E. Bron, U. König, G. Nénert, *Powder Diffr.* **2014**, *29*, S13–S18.

[6] F. Neese, *WIREs Comput. Mol. Sci.* **2012**, *2*, 73–78.

[7] T. Lu, F. Chen, *J. Comput. Chem.* **2012**, *33*, 580–592.

[8] W. Humphrey, A. Dalke, K. Schulten, *J. Mol. Graph.* **1996**, *14*, 33–38.
